# Proteome-Wide Analysis and Diel Proteomic Profiling of the Cyanobacterium *Arthrospira platensis* PCC 8005

**DOI:** 10.1371/journal.pone.0099076

**Published:** 2014-06-10

**Authors:** Sabine Matallana-Surget, Jérémy Derock, Baptiste Leroy, Hanène Badri, Frédéric Deschoenmaeker, Ruddy Wattiez

**Affiliations:** 1 Department of Proteomics and Microbiology, Interdisciplinary Mass Spectrometry Center (CISMa), University of Mons, Mons, Belgium; 2 Unit of Microbiology, Expert Group Molecular and Cellular Biology, Institute for Environment, Health and Safety, Belgian Nuclear Research Centre (SCK CEN), Mol, Belgium; Lawrence Berkeley National Laboratory, United States of America

## Abstract

The filamentous cyanobacterium *Arthrospira platensis* has a long history of use as a food supply and it has been used by the European Space Agency in the MELiSSA project, an artificial microecosystem which supports life during long-term manned space missions. This study assesses progress in the field of cyanobacterial shotgun proteomics and light/dark diurnal cycles by focusing on *Arthrospira platensis*. Several fractionation workflows including gel-free and gel-based protein/peptide fractionation procedures were used and combined with LC-MS/MS analysis, enabling the overall identification of 1306 proteins, which represents 21% coverage of the theoretical proteome. A total of 30 proteins were found to be significantly differentially regulated under light/dark growth transition. Interestingly, most of the proteins showing differential abundance were related to photosynthesis, the Calvin cycle and translation processes. A novel aspect and major achievement of this work is the successful improvement of the cyanobacterial proteome coverage using a 3D LC-MS/MS approach, based on an immobilized metal affinity chromatography, a suitable tool that enabled us to eliminate the most abundant protein, the allophycocyanin. We also demonstrated that cell growth follows a light/dark cycle in *A. platensis*. This preliminary proteomic study has highlighted new characteristics of the *Arthrospira platensis* proteome in terms of diurnal regulation.

## Introduction

*Arthrospira platensis*, better known as “*spirulina*”, is a motile non-heterocystous, non-N(2)-fixing cyanobacterium, that forms of a multicellular coiled filament, and it is typically found in alkaline lakes [1]. *Arthrospira* species provide exceptional nutritional value with high protein content (50–70% of its dry weight), it is rich in essential fatty acids, produces a variety of minerals, vitamins, and nutritional pigments such as phycocyanin [2] and thus has been proposed as a potential tool to manage the problem of malnutrition in developing countries. The *A. platensis* PCC 8005 strain was selected by the European Space Agency (ESA) for long-term space missions as a primary oxygen producer and also as an accessory balanced food provider for human crew survival in its Micro-Ecological Life Support System Alternative (MELiSSA) [3]. Therefore, proteomic studies of MELiSSA organisms appeared essential to complete the global behavior profile of these microorganisms in certain culture conditions.

The analysis of cyanobacterial proteins has been traditionally conducted using the proteome, primarily employing electrophoresis-based approaches [4]–[12]. Shotgun proteomics analysis involves the use of multidimensional protein/peptide separation to fractionate complex protein/peptide mixtures, thus simplifying the peptide samples for LC-MS/MS and enabling acquisition of MS/MS spectra for lower abundance peptides. The value of the use of such multi-faceted workflows was first demonstrated on the proteome of *Synechocystis* sp. PCC 6803 by Gan and co-workers [13]. Here, we report a shotgun proteomics study of *A. platensis* using a combination of LC-MS/MS approaches with gel-free and gel-based protein/peptide fractionation steps, such as one-dimensional gel electrophoresis (SDS-PAGE and IEF), 2D and 3D LC-MS/MS (Cu-IMAC) of the soluble and/or membrane and secreted protein fractions. Whole-genome sequencing of strain PCC 8005 and its annotation has been recently completed, and thus provide key assets to facilitate proteomics approaches [14]. Our present work adds new perspectives in the field of shotgun proteomics by developing a new fractionation method (Cu-IMAC) allowing the depletion of the most abundant proteins (phycocyanins) and enabling the detection of low abundance proteins. Another key point to increase the proteome coverage of *A. platensis* consists of simply diversifying experimental growth conditions, such as cultivating the cells under different light conditions, *i.e*. shifting from continuous light to a 12-hour light/dark (LD) cycle. This latter observation remains obvious but is still, unfortunately, too often ignored. This could represent a ‘biological fractionation' performed by the organism itself, with all the proteins not being constitutively expressed or being exposed to the same conditions [15].

Cyanobacteria are the simplest organisms in which circadian rhythms have been clearly documented [16], [17], most notably in the unicellular cyanobacterium *Synechococcus elongatus* [18]. Prediction of the performance of *A. platensis* under optimal growth conditions for optimal biomass and oxygen production and protein synthesis is crucial when one is considering large-scale production of the organism for space missions. For this purpose, we investigated how diel periodicity would influence cell growth and protein expression in *A. platensis*. This paper presents the first study on how *A. platensis* responds to being shifted from continuous lighting to a 12-hour LD cycle. Previously, mostly microarray methods have been applied to study the circadian clock at the transcript level [19], [20], [21], [22], however translation could also occur in a periodical manner. We report on the results of whole proteome profiling of the LD transition within the cyanobacterium *Arthrospira platensis*, using the ICPL post-digest labeling method that has been developed and optimized by our research group [23].

## Materials and Methods

### Strain and growth condition

*Arthrospira platensis* strain PCC 8005, obtained from the Pasteur Culture Collection of Cyanobacteria (PCC) was grown aerobically at 30°C under illumination by 100 µE m^−2^ s^−1^ in a rotary shaker (120 rpm) in 100 mL of modified Zarrouk's medium [24] until the mid-exponential growth phase was reached. The growth was monitored by optical density (OD) measurements at 620 nm (**λ** corresponding to the peak absorbance of phycocyanin). After one week of growth, cells from 100 mL of cultures were harvested at the mid-exponential phase (OD_620nm_≈0.7) by centrifugation at 8 000 *g* for 15 min at 4°C and the resulting pellet was washed with 50 mM phosphate buffer saline, pH 7.2 (Buffered saline pack, Pierce) and kept at −80°C until use. Larger culture volumes were required for the secretome analysis, and thus the cyanobacteria were cultured in 500 mL modified Zarrouk's medium. The diel rhythm experiments were conducted within an *Angelantoni scientifica* climate room maintained at 30°C, with a 12-hour light/dark transition growth cycle. After two pre-cultures to ensure synchronization to the LD cycle, the cell growth was monitored by optical density at 620 nm and cyanobacteria were harvested at their mid-exponential phase after both 12-hour light and 12-hour dark periods (OD_620nm_ = 0.8). Two separate experiments were performed for the diel cycle study.

### Shotgun and quantitative proteomics

#### Gel-based approach

For the peptide IPG-IEF separation, tryptic peptides were separated over an immobilized pH gradient (IPG) using isoelectric focusing (IEF) as described previously by Mastroleo and coworkers [25] with some modifications. Pelleted cells obtained from 100 mL culture were re-suspended in 1 mL lysis buffer (10 mM HEPES, pH 8; 6 M urea; 2 M thiourea). Protein samples were obtained by high power sonication (U50 control, IKA labortechnik, Germany) of the washed bacterial pellet suspended in one pellet volume of lysis buffer solution with 5 cycles of 10 s on ice at an amplitude of 60% and 1 pulse rate. Subsequently, samples were centrifuged at 13 500 rpm at 4°C, for 15 min. Protein samples were then reduced with 10 mM DL-dithiothreitol and alkylated with 55 mM iodoacetamide. A total of 600 *µ*g was digested overnight with trypsin (Promega, Belgium) at an enzyme/substrate ratio of 1/50 at 37°C, and the urea/thiourea concentration was reduced to 2 M before trypsin digestion by dilution with 10 mM NH_4_HCO_3_. The digested proteins were then desalted using HyperSep SpinTip C18 (Thermo electron) according to the manufacturer's instructions. The sample was evaporated to dryness in a vacuum centrifuge (Heto, Drywinner, Denmark) and resuspended in 700 *µ*L of 8 M urea supplemented with a trace of bromophenol blue. Digested proteins (300 *µ*g) were used to passively rehydrate both linear pH 3–10, pH 4–7, and pH 6–9 18 cm IPG strips (GE Healthcare) overnight. Isoelectric focusing was conducted on a Protean IEF cell (Bio-Rad) with a current limit of 50 *µ*A per strip at 20 °C with a 5-phase gradient program: 300 V for 1 h, a gradient 300–1000 V gradient for 1 h, 1000–4000 V for 3 h, 4000–8000 V for 3 h and the last phase was maintained until 100 000 V/h was reached. This last step lasted approximately 9 h. The gel strips were then cut in 35 equal length pieces. Peptides were extracted from each fraction by incubation in 100 *µ*L of 0.1% (v/v) formic acid for 1 h at room temperature. The extraction was repeated twice and subsequently combined with the initial fraction. Combined peptide extracts from each fraction were concentrated in a vacuum centrifuge (Heto, Drywinner, Denmark) to approximately 25 *µ*L. Each peptide fraction was desalted using Hypersep ZipTip C18 tips (Thermo electron) following the manufacturer's instructions and was subjected to LC-MS/MS analysis.

For the one-dimensional sodium dodecyl sulfate-polyacrylamide gel electrophoresis (SDS-PAGE) approach, the bacterial pellets were resuspended in Phosphate Buffer Saline (50 mM) (Pierce, Belgium). Cells were lysed by three passages through a French Press (Thermo, Belgium) at 500 psi. After ultracentrifugation at 40 000 rpm at 4°C for 40 min, the supernatant (soluble proteome) and pellet (membrane proteome) were saved for SDS-PAGE separation. The supernatant and the membrane pellet were diluted or resuspended in Laemmli buffer (2% SDS, 10% glycerol, 5% *β*-mercaptoethanol, 0.002% bromophenol blue and 0.125 M Tris-HCl, pH 6.8) and sonicated in a water bath six times for 1 min at room temperature. After 1 min incubation at 90°C, the protein solutions were centrifuged at 13 000 rpm at room temperature for 15 min. The SDS-PAGE of the soluble and membrane protein mixtures was conducted using 4–20% precast polyacrylamide mini-gels (Pierce). The protein bands were visualized with staining using the Imperial Protein Stain (Thermo) according to the manufacturer's instructions. The corresponding gel lane containing proteins was cut in 30 pieces of 1 mm each. Enzymatic digestion was performed by the addition of 10 *µ*L modified sequencing grade trypsin (0.02 mg/mL) (Promega) in 25 mM NH_4_HCO_3_ to each gel piece. The samples were placed for 15 min at 4°C and incubated overnight at 37°C. The reaction was stopped with 1 *µ*L 5% (v/v) formic acid. Tryptic peptides were analyzed by LC-MS/MS.

For the secretome fraction, a 500 mL-culture was centrifuged at 8 000 g for 15 min at 4°C and then filtered through a 0.2-µm filter to remove any suspended cells and transferred into a 3 kDa cutoff Amicon Ultra-15 filter unit (Millipore) to be concentrated into a smaller volume (∼5 mL). Subsequently, the concentrated proteins were precipitated using 40% (w/v) of trichloroacetic acid. The resulting pellet was resuspended in Phosphate Buffer containing 2 M urea and separated by SDS-PAGE, as described above.

#### Gel-free approach

**Figure 1:**
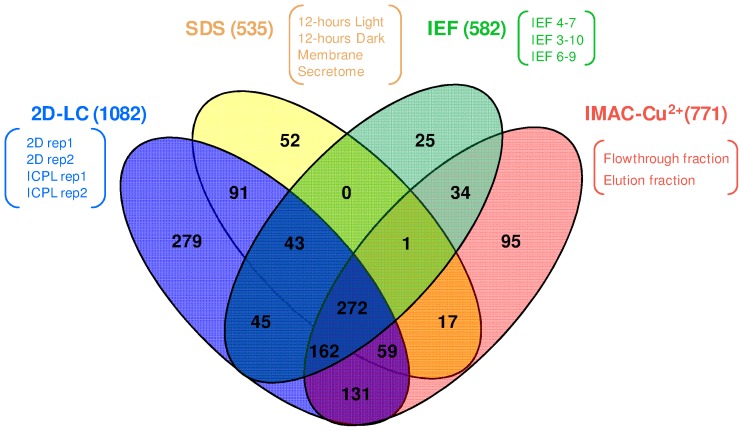
Venn diagram showing non-redundant proteins identified using: IEF, SDS-PAGE, 2D-LC, and 3D-LC MS/MS (IMAC-Cu^2+^).

Sonication and sample clearing by centrifugation was performed as mentioned above except the lysis buffer used here was a guanidine chloride solution (6 M). Proteins samples were reduced with 5 mM Tris (2-carboxyethyl) phosphine at 60 °C for 30 min and alkylated with 0.4 *µ*M iodoacetamide at 25 °C for 30 min. Proteins were recovered by acetone precipitation (1 h) with an acetone/protein ratio of 4/1. After 15 min centrifugation at 13 000 rpm and acetone evaporation, the resulting pellet was dissolved in 100 mM phosphate buffer (pH 8) containing 2 M urea. Total protein concentration was determined using the Bradford method, according to the Bio-Rad Protein Assay kit (Bio-Rad, Hertfordshire, UK) according to manufacturer's instructions, with bovine *γ*-globulin as a protein standard. Overnight enzymatic digestion was carried out with modified sequencing grade trypsin (Promega) at an enzyme/substrate ratio of 1/25 at 37°C. Tryptic peptides were separated by 2D LC-MS/MS. 2D LC experiments were conducted 4 times with 2 biological replicates (2 runs for identification without ICPL labeling: 2D rep1/2 and 2 runs for quantification with ICPL labeling: ICPL rep1/2; Figure 1).

#### Post-digest ICPL labeling

For the differential proteomic analysis of light and dark-cultured cells, proteins were extracted and tryptic peptides were prepared as described in the gel-free approach section. The proteins extracted from the light- and dark-cultured cells were labeled according to the post-digest ICPL labeling procedure, recently described and optimized by Leroy and colleagues [23]. Briefly, after enzymatic digestion, 33 µg tryptic peptides were submitted for labeling using 3 µL ICPL-labeling reagent for 90 min at room temperature. This first labeling step was followed by the addition of 1.5 µL supplemental reagent and the reaction was allowed to proceed for 90 additional min. The protein samples obtained from the dark grown cells were labeled with 6-[^12^C] nicotinoyl-*N*-hydroxysuccinimide (light label), while the mixtures from light growth states were labeled with 6-[^13^C] nicotinoyl-*N*-hydroxysuccinimide (heavy label). After removing any excess of reagents according to the manufacturer's instructions, the two samples were combined, and the resulting labeled peptide labeled mixture was basified with 2 µL 2N NaOH and subsequently 2 µL 2N HCl. After desalting using HyperSep C18 SpinTips, 40 µg digested proteins were analyzed by 2D LC-MS/MS. ICPL experiments were conducted twice with 2 different biological replicates.

#### Cu (II)-IMAC prefractionation

Proteins extract was obtained from bacterial culture using a French Press as described in the SDS-PAGE approach. The protein solution was then incubated with 20 mM EDTA for 5 min at room temperature and subsequently dialyzed against phosphate buffer (20 mM, pH 7, 0.5 M NaCl) at 4°C. One milliliter fast-flow Sepharose gel containing iminodiacetic acid group (Amersham Pharmacia) was immobilized with 0.2 M CuCl_2_ in phosphate buffer (20 mM, pH 7, 0.5 M NaCl). Excess copper (II) ions were removed by washing the column with 0.1 M sodium acetate, 0.5 M sodium chloride (pH 3.8) and then equilibrated with 20 mM sodium phosphate, 0.5 M sodium chloride (pH 7). A total of 1 mg protein extract solubilized in phosphate buffer containing 0.5 M NaCl was applied onto the column and the flowthrough was collected in 1-mL fractions. The absorbance at 280 nm of each fraction was measured with an Analytical UV Detector (Pharmacia LKB-Unicord SII), and the flow rate was controlled by a peristaltic pump (Pharmacia LKB Pump P-1) at 1 mL min^−1^. The bound proteins were eluted using 20 mM imidazole. Subsequently, 45 µg protein from the flowthrough and bound fractions were subjected to reduction, alkylation, trypsinolysis and analysis by 2D LC-MS/MS, as described previously in the gel-free approach. The methodology using the Cu (II) IMAC separation will be hereafter referred to as 3D LC-MS/MS.

### Liquid Chromatography Tandem Mass Spectrometry (LC-MS/MS)

Regarding the samples obtained from the gel-based approaches (IPG-IEF and SDS-PAGE), purified peptides from digested protein samples were identified using an LC Ultimate 3000 system (Dionex) coupled with an HCT ultra plus mass spectrometer (Bruker Daltonics, Germany). Six microliters of each fraction were loaded onto a pre-column (C18 Trap, 300 *µ*m i.d.×5 mm, Dionex) using the Ultimate 3000 system delivering a flow rate of 20 *µ*L/min loading solvent (5% (v/v) acetonitrile (ACN), 0.025% (v/v) TFA). After a 10 min desalting step, the pre-column was switched online with the analytical column (75 *µ*m i.d.×15 cm PepMap C18, Dionex) equilibrated in 96% solvent A (0.1% (v/v) formic acid in HPLC-grade water) and 4% solvent B (80% (v/v) ACN, 0.1% (v/v) formic acid in HPLC-grade water). Peptides were eluted from the pre-column to the analytical column and then to the mass spectrometer with a gradient from 4 to 57% solvent B for 50 min and 57–90% solvent B for 10 min at a flow rate of 0.2 µL min^−1^ delivered by the Ultimate pump. The separated peptides were analyzed online using an ion-trap mass spectrometer. Positive ions were generated by electrospray and the instrument was operated in an information-dependent acquisition mode described as follows: MS scan range: 300–1500 *m*/*z*, maximum accumulation time: 200 ms, ICC target: 200 000. The top 4 most intense ions in the MS scan were selected for MS/MS in dynamic exclusion mode: ultrascan, absolute threshold: 75 000, relative threshold: 1%, excluded after spectrum count: 1, exclusion duration: 0.3 min, averaged spectra: 5, and ICC target: 200 000.

For the 2D LC-MS/MS approach, dried proteins samples were resuspended in 8 µL of the loading solvent. Six microliters containing 40 µg proteins were loaded onto a strong cation exchange (SCX) column (POROS S10, 10 cm, Dionex) and eluted sequentially using 5, 10, 25, 50, 75, 100, 120, 150, 200, 400, and 800 mM sodium chloride (20 µL). The unbound fraction and solution from each salt step were concentrated and desalted on a C18 pre-column (C18, 15 cm, i.d. 75 *µ*m, Dionex) at 20 µL min^-1^. After a 15 min wash with the loading solvent, the pre-column was switched inline with an analytical column containing C18 reverse-phase packing material (Dionex). Peptides were eluted using a linear gradient of CH_3_CN at 0.2 µL min^−1^. The ACN gradient was 4–37% solvent B (solvent B: 80% ACN, 0.08% formic acid) in 100 min, 37–57% solvent B in 10 min and 57–90% solvent B in 10 min; 90% solvent B was maintained for 10 min and then reset to 4%.

### Data processing

All MS/MS spectra were searched against an *A. platensis* PCC8005 protein database (retrieved from MaGe Annotation Platform, *updated genome version 3*, 6355 proteins) [14] using Mascot (Matrix Science, London, UK; version 2.2) with fragment precursor and product ion tolerances of ±1.3 and an MS/MS tolerance of ±0.5 Da with the maximum number of missed trypsin cleavage sites set to 1. Oxidation of methionine, iodoacetamide derivatization of cysteine (Cys-carbamidomethyl) and for the quantitative proteomics samples, post-digest ICPL quantification chemistry of lysine and the peptide N-terminus were specified in Mascot as variable modifications. High scores indicated a likely match. All matching spectra of the proteins with a Mascot score greater than 25 and lower than 50 were manually inspected to ensure ion progressions of at least 4 consecutive y- or b-type ions, especially for the proteins identified with one single peptide. Any matches that did not meet these criteria were rejected. A first step of normalization based on the median of the peptide mass ratios was performed on Mascot distiller. The protein ratios correspond to the average of peptide ratios. Only peptides with an ion score above 30 were considered for quantification. The false discovery rate (FDR) was calculated at the peptide level for all experimental runs using the Decoy option in Mascot; this rate was estimated to be lower than 1% for gel-based methods and lower than 2% for gel-free methods using the identity threshold as the scoring threshold system.

The relative quantification of proteins was calculated based on the intensity ratio of isotope-labeled peptide pairs (light and heavy stable isotope, corresponding to dark and light cultured cells, respectively). MS/MS spectra were searched against the *A. platensis* database for protein quantification using Mascot distiller v.2.3.2 (Matrix Science, London, UK) and the search settings were similar to those used for Mascot. The global mean and SD of the protein ratios were calculated for each sample pair. Peak lists (.baf files) from the ESI-TRAP data were generated using Mascot Distiller (Version 3.2.1.0, Matrix Science). Parameters for the quantification procedure using Mascot Distiller were as follows: correlation threshold: 0.8; integrated method: Simpsons; XIC threshold: 0.2; max XIC width: 7; quality: fraction.

To determine reliable quantitative data, manual validation of all quantified peptides was performed to filter out co-eluted peptides and/or noise. Moreover, a quantified protein was retained only if at least two well-identified peptides were provided. The average and geometric standard deviation of ICPL ratios for the proteins identified in the two biological replicates were calculated in log space, converted back and represented in linear space. Data were recorded for proteins with an average increased abundance above 1.25 or below 0.8 relative to the dark phase sample, and data were distinguished as significantly different from unity by bold characters (Table 1) using the following comparison test provided by Mascot Distiller:
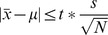
where N is the number of peptide ratios, *s* is the standard deviation and *x* the mean of the peptide ratios. Both numbers were calculated in log space. *t* is the value of Student's t test for *N-1* degrees of freedom and a two-sided confidence level of 95%. The emPAI values provided by Mascot software were used as an indication of relative abundance which takes into account the number of sequenced peptides per protein and has been shown to be directly proportional to protein content [26].

**Table 1:** Quantitative proteomics table of proteins with differential abundance (ratio below 0.8 or above 1.25 relative to the dark growth phase) in both biological replicates with their associated geometric standard deviation.

Accession number	Proteins name	ICPL Rep1			ICPL Rep2		
		Light/Dark Ratio	SD(geo)	#Pept	Light/Dark Ratio	SD(geo)	#Pept
ARTHROv3_190004	Phosphoenolpyruvate synthase	**0.43**	**1.22**	3	**0.55**	**1.30**	**4**
ARTHROv3_1130101	Conserved hypothetical protein	**0.43**	**1.09**	**2**	**0.53**	**1.45**	**4**
ARTHROv3_930103	Photosystem I P700 chlorophyll a apoprotein A2 (PsaB)	**0.52**	**1.68**	**7**	**0.52**	**1.39**	**8**
ARTHROv3_1050021	Isocitrate dehydrogenase [NADP]	**0.52**	**1.04**	**2**	**0.55**	1.13	2
ARTHROv3_1620020	Photosystem II P680 chlorophyll A apoprotein (CP-47 protein)	**0.66**	**1.56**	**9**	**0.42**	**1.65**	**5**
ARTHROv3_210030	Photosystem II 12 kDa extrinsic protein precursor (PS II complex 12 kDa extrinsic protein) (PSII-U)	**0.51**	**1.37**	**5**	**0.67**	**1.23**	**3**
ARTHROv3_930104	Photosystem I P700 chlorophyll a apoprotein A1 (PsaA)	**0.66**	**1.45**	**7**	**0.59**	**1.76**	**8**
ARTHROv3_810100	Hypothetical protein	0.62	1.24	2	**0.67**	**1.04**	**2**
ARTHROv3_6720001	Photosystem Q(B) protein 1 precursor (Photosystem II protein D1 1)	**0.72**	**1.31**	**4**	**0.61**	**1.39**	**6**
ARTHROv3_1210006	Photosystem II CP43 protein (PSII D2 protein)	**0.74**	**1.27**	**5**	0.53	1.40	4
ARTHROv3_1620014	Acyl carrier protein	0.76	1.24	2	**0.60**	**1.08**	**4**
ARTHROv3_630063	ATP synthase B chain (Subunit I)	**0.66**	**1.30**	**5**	0.73	1.36	3
ARTHROv3_810066	Putative Calvin cycle regulator CP12-like protein	0.72	1.44	4	**0.70**	**1.31**	**5**
ARTHROv3_630062	ATP synthase delta chain; ATP synthase F1. delta subunit	**0.65**	**1.18**	**4**	0.81	1.27	7
ARTHROv3_810107	Phosphoenolpyruvate synthase (part 2)	**0.88**	**1.84**	**16**	**0.67**	**1.71**	**16**
ARTHROv3_2890001	Protein chain elongation factor EF-G. GTP-binding	**0.76**	**1.22**	**8**	0.79	1.31	6
ARTHROv3_760043	Conserved hypothetical protein	**0.81**	**1.14**	**5**	**0.78**	**1.14**	**6**
ARTHROv3_1100012	Phycobilisome 32 kDa linker polypeptide. phycocyanin-associated. rod 1	1.13	1.31	10	**1.30**	**1.36**	**14**
ARTHROv3_5910003	Allophycocyanin alpha subunit	**1.34**	**1.33**	**13**	1.14	NN	10
ARTHROv3_1400046	Inositol-5-monophosphate dehydrogenase	**1.49**	**1.39**	**4**	1.15	1.51	4
ARTHROv3_6740003	30S ribosomal protein S1	1.41	1.65	7	**1.33**	**1.22**	**8**
ARTHROv3_1350003	Hypothetical protein	1.28	1.25	5	**1.43**	**1.11**	**5**
ARTHROv3_680013	Hypothetical protein	**1.37**	**1.26**	**7**	**1.41**	**1.26**	**8**
ARTHROv3_1100014	Phycobilisome 8.9 kDa linker polypeptide. phycocyanin-associated. rod (Rod-capping linker protein)	**1.37**	**1.28**	**6**	1.42	NN	9
ARTHROv3_1610009	Adenylosuccinate synthetase. IMP—aspartate ligase. succinoadenylic kinosynthetase	**1.37**	**1.12**	**2**	**1.57**	**1.01**	**2**
ARTHROv3_1280008	Allophycocyanin beta-18 subunit	1.51	1.98	4	**1.49**	**1.35**	**6**
ARTHROv3_860009	30S ribosomal protein S6	1.34	1.24	4	**1.71**	**1.64**	**6**
ARTHROv3_1540080	30S ribosomal subunit protein S4	**1.81**	**1.11**	**2**	1.55	1.34	4
ARTHROv3_310008	30S ribosomal subunit protein S21	**1.68**	**1.24**	**4**	**1.89**	**1.02**	**2**
ARTHROv3_870040	Glycine-rich RNA-binding protein. rbp-like	3.43	2.89	7	**4.12**	**2.36**	**5**

Their significant difference from the unity was distinguished by using bold characters.

### Pigments analysis

The pigments of phycocyanin and chlorophyll were measured for cells harvested 30 min before the transition from light to dark and dark to light phase as well as 1 hour after the transition to the light and dark phases. Samples were stored immediately at −80°C after harvesting. Pellets were freeze dried overnight, and each fraction was weighted to obtain the same absolute dry weight. Cells were lysed by resuspending the pellets in 0.05 M Na_2_HPO_4_, pH 7, and five freeze/thaw cycles were performed to extract the fraction of phycobiliprotein containing the phycocyanin pigments. Subsequently, an additional treatment with lysozyme (50 mg/mL) for 30 min at 37°C was applied to perform total cell extraction. The supernatant was then measured using a spectrophotometer at wavelengths 615 and 652 nm and the concentration of phycocyanin fraction was calculated.

The optical density of the supernatant was measured at 615 and 652 nm using an UV-VIS spectrophotometer (Elico). Phycocyanin concentration (PC) was calculated according to Bennett and Bogorad [27]: 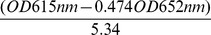



Regarding the chlorophyll extraction, the same dried pellets of *A. platensis* were washed three times with 0,05 M Na_2_HPO_4_, pH 7 buffer, treated with pure methanol, and then sonicated (30% amplitude, 60 sec, 1 pulse) for cell lysis. The supernatant containing the chlorophyll was measured using a spectrophotometer at an OD_665_ nm, and the concentration was calculated according to the extinction coefficient of methanol 100%: 74.5 mL mg^−1^cm^−1^ [27].

## Results

### Comparison of the different experimental approaches used in the shotgun proteomics workflow

A total of 1306 proteins were identified by LC-MS/MS, corresponding to 21% of the theoretical proteome of *A. platensis* PCC 8005. Throughout the text, the expression of “theoretical proteome” will refer to the total potential set of proteins predicted from the genome sequence of *A. platensis* PCC8005 deduced from the recent genome sequence analysis we performed [14], whereas the proteins experimentally identified by mass spectrometry will be referred to as the “expressed proteome”. Our general experimental strategy consisted of diversifying proteins fractionation methods in order to increase the predicted proteome coverage of *A. platensis*. The gel-free approaches, namely 2D and 3D LC-MS/MS, provided the highest number of identified proteins: 1082 and 771 proteins, respectively (Figure 1). The gel-based approaches, namely IPG-IEF- and SDS-PAGE-LC MS/MS, resulted in a similar number of identifications, with 582 proteins and 535 proteins, respectively. When comparing all gel-based and gel-free approaches in terms of non-redundant identified proteins, a relatively high overlap between the techniques was observed. A significant number of proteins were only detected in 2D or 3D LC-MS/MS analyses, with 279 and 95 specific proteins detected, respectively. Conversely only 25 and 52 proteins were found to be specific to the gel-based IPG-IEF and SDS-PAGE approaches (Figure 1). Interestingly, all the soluble proteins identified with the SDS-PAGE method were also characterized by using the gel-free approaches, and thus the 52 proteins specific to this method were either associated with the isolation protocol (membrane or secreted proteome) or a specific cell growth condition (12-hours LD cycle). This latter result emphasizes the fact that increased proteome coverage can be reached by using multiple protein extraction protocols as well as the overall set of cell growth conditions. Protein secretion is required for numerous aspects of the microorganism's life cycle. The bacterial signaling network includes a wide diversity of interacting proteins that monitor environmental and intracellular parameters. A study on the cyanobacterium *Synechocystis* previously reported the spectrum of secreted proteins [28]. In total, seven N-terminal sequences were identified and the major ones corresponded to cyanobacterial pilin, PilA. In our study, a total of 10 secreted proteins were identified with Mascot but only 4 of them remained in the dataset after MS/MS data manual validation: Diguanylate cyclase/phosphodiesterase with PAS/PAC sensor(S) (ARTHROv3_1620004), recA DNA strand exchange and recombination protein with protease and nuclease activity (ARTHROv3_1050022), Ribosomal biogenesis GTPase; GTP-binding protein HSR1-related (ARTHROv3_1300029), and the Transposase IS200/IS605 family (OrfB) (ARTHROv3_230006). Only one of those latter proteins - the ribosomal biogenesis GTPase (ARTHROv3_1300029) was specific to the secretome and not characterized in any of the other conditions/methods (Table S1). Diguanylate cyclase/phosphodiesterase protein containing GGDEF/EAL domains are encoded in the majority of bacterial genomes [28]. These proteins have been implicated in the biosynthesis of exopolysaccharides, formation of biofilms, establishment of a sessile lifestyle, surface motility, and regulation of gene expression [29]. Proteins involved in DNA strand exchange (ARTHROv3_1050022) and transposase (ARTHROv3_230006) were characterized and could be engaged in horizontal gene transfer in *A. platensis*. More experiments in regards to this very preliminary secretome analysis should be performed to obtain a higher number of identified proteins toward the thorough elucidation of their role in cell-cell signaling in *A. platensis*.

**Figure 2:**
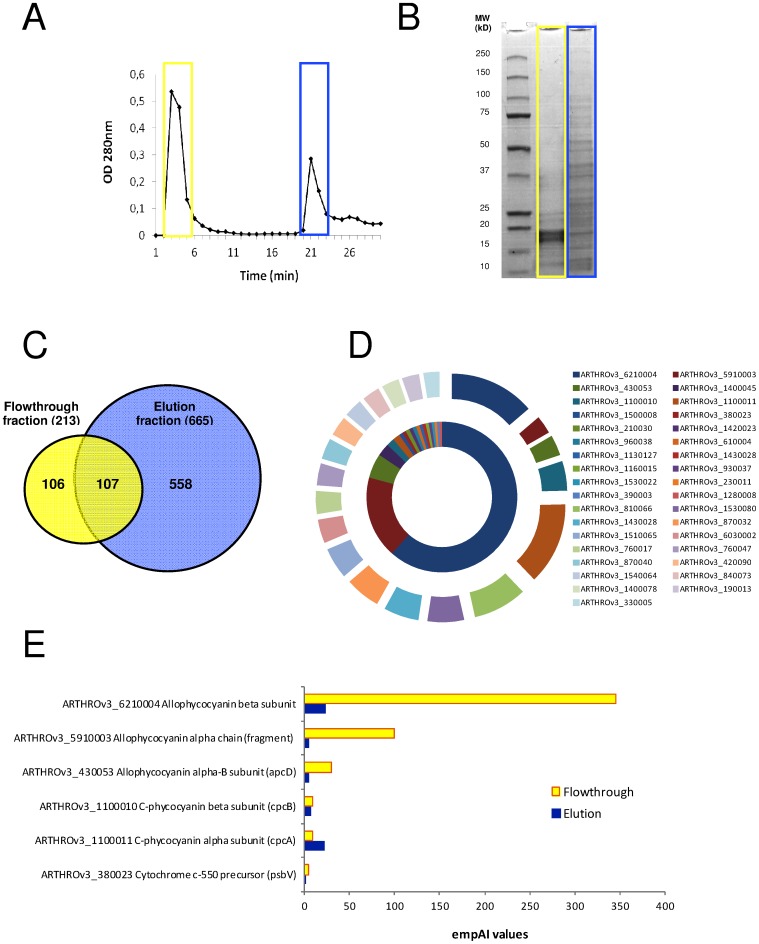
IMAC-Copper chromatogram (A). 4-20% SDS PAGE (B). Venn diagram showing non-redundant proteins between the two fractions (C). The twenty highest emPAI values in both the flowthrough (inner circle) and elution (outer circle) fractions (D). Histogram representing the emPAI values of the 6 most abundant proteins found to be common between the flowthrough and eluate (E).

The total number of identified proteins using a single 3D LC-MS/MS run (771 proteins combining the flowthrough and elution fractions) was much higher than the number obtained from a single 2D LC-MS/MS run (average of 578 proteins) (Table S1). It is important to note that four different 2D LC-MS/MS analyses were performed *vs* against only one 3D LC-MS/MS experiment, thus explaining the difference in the total number of characterized proteins with 1082 and 771 proteins, respectively (Figure 1). Figure 2A presents the IMAC chromatogram exhibiting one non-retained fraction (yellow box) and one retained fraction (blue box). Those two fractions showed different profiles on SDS-PAGE, with a larger number of proteins bands for the elution fraction (blue box) than the flowthrough (yellow box) (Figure 2B). The 2D LC-MS/MS analysis of the non-retained and retained fractions confirmed the latter results, with a total of 213 identified proteins against 665 proteins, respectively (Figure 2C; see also Table S2 and Table S3 for a complete list of the proteins identified in the flowthrough fraction and the eluate, respectively). The most striking result was the efficiency of the IMAC-Copper technology to eliminate the phycocyanins, the most abundant proteins and thus enabling the detection of less abundant proteins. The emPAI values permitted us to estimate the relative protein amount of *A. platensis*, subsequently to the IMAC-Copper method. In this approach, when comparing the emPAI values of the two fractions, we observed that among the 20 more abundant proteins (providing the highest emPAI score) only 5 were determined to be shared between the two fractions (ARTHROv3_6210004; ARTHROv3_430053; ARTHROv3_1100010; ARTHROv3_1100011; ARTHROv3_1430028; Figure 2D; see also Table S4 for a complete list of the common proteins found in both fractions). The top 5 most abundant proteins of the flowthrough fraction were comprised of 4 phycocyanins and a cytochrome c precursor (Figure 2E). The predominance of the most abundant proteins observed in the non-retained fraction was significantly decreased in the elution fraction, extensively lowering the amount of allophycocyanin alpha and beta subunit (ARTHROv3_5910003; ARTHROv3_6210004) by 20-fold and 15-fold, respectively.

### Differential protein abundance during diel cycle

**Figure 3:**
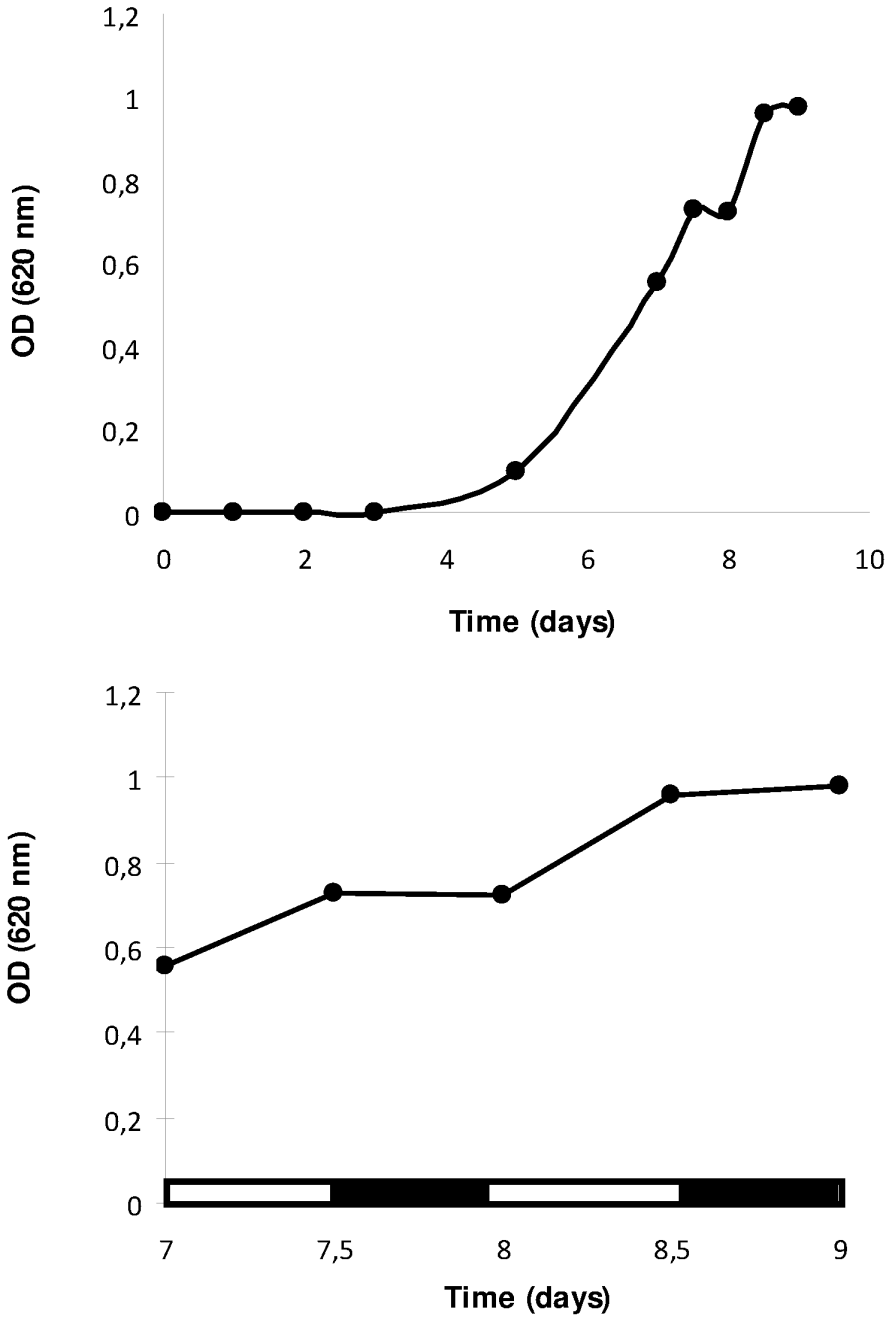
Growth curve of *A*. *platensis* under a 12-hour LD cycle.

**Figure 4:**
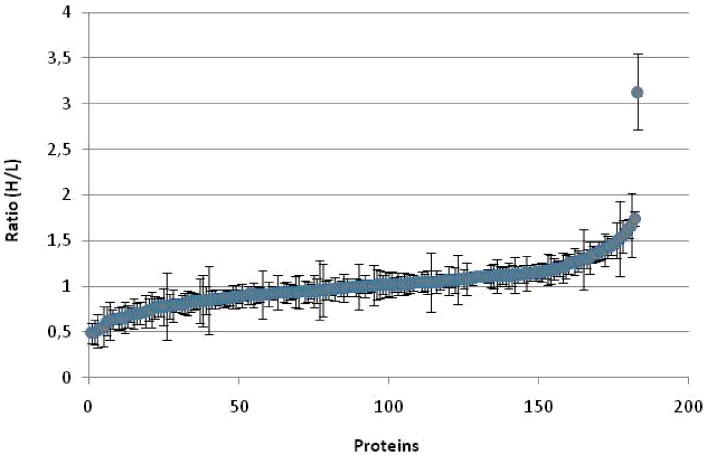
Distribution of the fold-changes of all proteins quantified (185) in both biological replicates of the LD growth cycle using the post-digest ICPL method.

**Figure 5:**
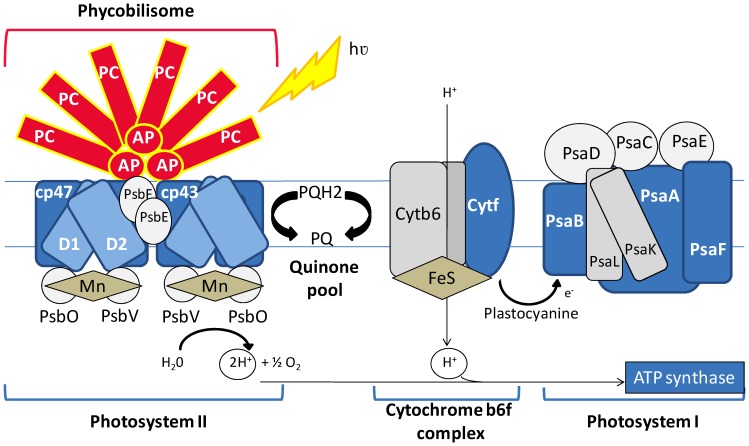
Cartoon depicting proteins associated with photosynthesis that are differentially regulated under the LD cycle. Each protein of the diagram is colored based on increased (red) or decreased abundance (blue) relative to the dark growth phase. Proteins colored in grey were not quantified.

**Figure 6:**
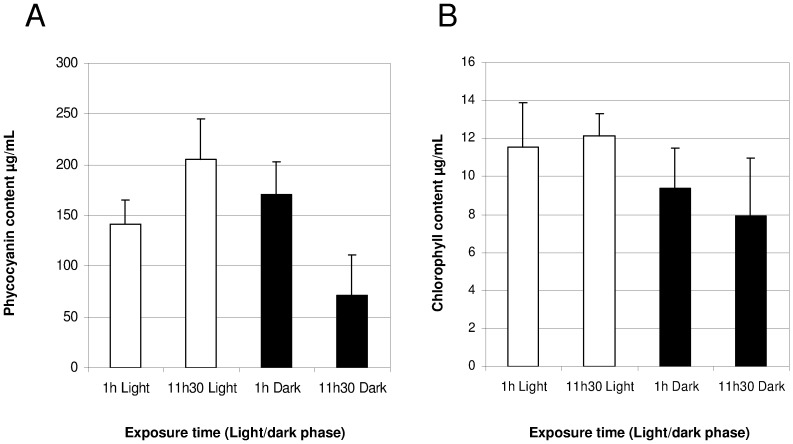
Phycocyanin (A) and chlorophyll content (B) according to two different time points of the light (white bars) and dark phases (black bars).

*A. platensis* was cultivated under different light conditions, shifting from continuous light to a 12-hour LD cycle and quantitative proteomics was assessed using post-digest ICPL labeling. Figure 3 shows the growth curve of *A. platensis* with an alternating LD cycle. Cells divided once per day, and this occurred cyclically most likely during the light phase, with an increase of biomass at the end of the light phase. Although quantitative data were obtained for 482 proteins, after manual inspection 309 ICPL post-digest-labeled proteins were found to be properly quantified. A total of 185 were identified in the two biological replicates, representing an overall coverage of 14% of the expressed proteome. The differential expression ratio of the 185 proteins ranged from 0.5 to 3 (Figure 4). For statistical analysis, only proteins quantified with at least two peptides and significantly different from unity were considered. After applying a commonly used threshold of 0.8 and 1.25, we observed a total of 30 proteins significantly differentially regulated under LD transition. A total of 17 and 13 proteins were found to be more abundant at the end of the light phase and dark phase, respectively (Table 1). Among the 30 proteins, the main function found to be mostly affected by the LD cycle appeared to be related to energy production with several proteins involved in photosynthesis that were found to be more abundant under the light phase compared to the dark phase (Table 1). Photosynthetic proteins controlled by the LD cycle are indicated in Figure 5, by using a color code based on their relative abundance level (down- or up-regulated: blue and red, respectively) which will be discussed below. The content of the phycocyanin and chlorophyll pigments were measured for the light to dark and dark to light transitions. As depicted in Figure 6, the content of phycocyanin was found to be modulated by the light or dark treatments while the amount of chlorophyll was found to be stable regardless of light/dark treatment (Figure 6).

## Discussion

### The power of extensive protein fractionation with a 3D LC-MS/MS methodology

The selective removal of the highly abundant proteins was found to be crucial to access additional low-abundance proteins. Accordingly, we demonstrated the efficiency of the additional fractionation step at the protein level according to the metal binding properties to increase the protein identification confidence and number. IMAC-Copper performed the best to isolate the most abundant phycobiliproteins among all the methods and it produced the greatest number of identified proteins (Table S1, Figure 2).

Immobilized metal affinity chromatography charged with Cu^2+^ was used to identify over 500 proteins with metal-binding properties. Combining classical immobilized ion affinity chromatography (IMAC) and modern proteomic techniques (2D LC-MS/MS) metal binding proteins have been successfully isolated and identified to define a metalloproteome. Approximately one quarter to one third of all proteins require metals [30], which represents over 1500 proteins in *Arthrospira platensis*, although the exploitation of the elements varies among cells. Metalloproteins are widespread in nature, comprise at least one metal ion and are involved in many diverse fundamental biological processes [30]. They are basically involved in electron transfer (cytochrome, photosystem II, plastocyanines) as well as catalysis (protease, carboxylase, hydorlase, superoxide dismutase). Copper is used in cytochrome oxidase, plastocyanin and several periplasmic enzymes [30]. In addition to amino acid sequence, protein surface characteristics and protein folding are additional parameters that determine metal-binding affinity. These affinities to divalent metals follow a universal order of preference known as the Irving Williams series (Mg^2+^ and Ca^2+^ weakest binding (Mn^2+^<Fe^2+^<Co^2+^<Ni^2+^<Cu^2+^>Zn^2+^) [31], [32]. According to this classification, all metalloproteins could bind tightly the most competitive metal, the divalent copper metal. A recent study showed that one copper protein and one manganese protein, both sharing similar cupin folds and the same protein ligands, preferred copper to manganese *in vitro*, consistent with the Irving Williams series [33]. Initial time-course studies of proteins binding to Cu^2+^-IMAC revealed that proteins bound rapidly and that 1 min incubations were sufficient to permit binding [33].

A novel aspect of this study is that 3D LC-MSMS was used to reduce the complexity of a total complex protein extract, allowing the examination of the copper binding behavior of several proteins. In our experiment, there are strong correlations between the sets of immobilized metal affinity chromatography-interacting proteins and the proteins predicted to contain metal-binding motifs. Interestingly, the list of Cu^2+^-binding proteins identified included a large number of proteins not previously known to bind copper and were diverse in function (Table S3). The reasons why several proteins were found to copurify with Cu-interacting proteins could be the result of (a) proteins harboring different divalent metal binding sites, (b) proteins containing histidine clusters on their surfaces or (c) proteins that bind copper binding proteins. We could thus explain the fixation of some of the allophycocyanin by the simple fact that it has been previously reported that the APC core interacts with the PSII at a region characterized by an electron-microscopy projection map [34]. Because of the small amount of co-eluting allophycocyanins, the 3D LC-MS/MS technology provided a suitable tool to eliminate the most abundant protein and thus to improve this cyanobacterial global proteome coverage.

### Diel cycle analysis

It was previously demonstrated in other cyanobacterial species that major steps in the cell cycle (DNA replication and cytokinesis) and key metabolic functions (e.g., oxygenic photosynthesis, nitrogen fixation, amino acid uptake) were restricted to specific times of the day [16], [17]. This internal mechanism of gene regulation, called circadian clock, coordinates physiological events with the external environment, allowing the cells to anticipate cyclic environmental changes. Light and temperature are the two environmental stimuli that are well known to synchronize the phase of the circadian rhythm with an oscillation that has an approximately 24 h period. It is important to distinguish circadian rhythms from simple light-dependent responses. A rhythm cannot be said to be endogenous, and called circadian until it has been shown to persist under constant environmental conditions (*i.e*., constant lighting) with a period of approximately 24 h. This is the only way to distinguish a circadian clock from diurnal rhythms that are simply a response to a 24 h environmental change. However, almost all diurnal rhythms are found to be circadian [35]. The circadian clock is controlled by three proteins KaiA, KaiB and KaiC that are essentials for the clock to function [18], [36]. Genome sequencing has revealed that *A. platensis* possesses the three genes KaiA, KaiB and KaiC (ARTHROv3_490006; ARTHROv3_490005; ARTHROv3_490004) and thus this cyanobacterium was expected to exhibit a light/dark periodicity. Interestingly, we confirmed the expression of these three genes in our shotgun study (Table S1). Unfortunately, the kaiC and kaiA proteins were identified in only one replicate (ICPL1) of the quantitative proteomic analysis within the diel study that consequently did not enable interpretation of the regulation of this gene (Table S1). It is important to emphasize here that the response of *A. platensis* to LD growth transition could be the result of either an endogenous clock or a light-dependent regulation. The "unique" identification of the ftsZ cell division protein (ARTHROv3_1420011) during the light phase and not in the dark phase is indicative of it possibly playing a role in the cell division controlled by the LD clock (Table S1).

In our experiment, growth of *A. platensis* was found to be under control of a light/dark oscillator with an increase of biomass occurring periodically at the end of the day (Figure 3). During the light phase, photosynthesis provides energy for cell growth and subsequently cell division could occur when cells reach an appropriate size. It was previously reported that, at the light/dark transition, cells were blocked in the S phase due to the presence of dark-block points before chromosome replication [37]. In this manner, darkness would inhibit new initiation of replication. Moreover, it has long been recognized that cyanobacterial metabolism becomes largely (but not totally) quiescent in the dark [38]. The cell division cycle is not regulated identically according to the cyanobacterial species [39]. Indeed, while both *A. platensis* and *S. elongatus* are undergoing cell division at the end of the day, *Prochlorococcus* cell division occurs during the night [39]–[41].

The day to night transition is also marked by a switch in energy metabolism from photosynthesis to aerobic respiration. It is thus not surprising that the photosynthetic capacity varies along the diel cycle. Accordingly, *A. platensis* was found to have rhythms in its photosynthetic activity. Cyanobacteria photosynthesis is performed on thylakoïd membranes located in the border areas of cytoplasm. As depicted in Figure 5, the photosystem (PS) includes PSI and PSII functionally linked to each other: one splits H_2_O into O_2_ and the other reduces NADP to NADPH. In our study, five PSII reaction center proteins (ARTHROv3_1620020; ARTHROv3_210030; ARTHROv3_6720001; ARTHROv3_1210006; ARTHROv3_1310019) and three PSI reaction center proteins (ARTHROv3_930103; ARTHROv3_930104; ARTHROv3_1490027) were likewise cycling with a L/D ratio of approximately 0.6. Note that the two proteins, namely cytochrome f (ARTHROv3_170006) and the Photosystem I reaction center subunit III precursor (ARTHROv3_1490027) were not listed in Table 1, because their ratios (L/D = 0.6) were not significantly different from unity; however, those proteins were well quantified in both replicates (Data S1). Accordingly, they are represented in blue in Figure 5 to confirm the down-regulation tendency of photosynthesis-related proteins. It is interesting to note that *A. platensis* like *S. elongatus* possesses several genes of *psbA* genes encoding the protein D1 from PSII (*psbA1*, *psbA2*, *psbA3*, *psbA4*) and only one D1 protein, encoded by the *psbA1* gene was found to be down-regulated upon a light/dark cycle (ARTHROv3_6720001; 0.65). Notably, it appeared that almost all quantified proteins associated with PSII and PSI were down-regulated in the light phase (Figure 5; Table 1). However, it is noteworthy that the abundance of proteins can change not only as a result of gene expression but also by increasing protein stability and turnover. Hence, the similar protein ratios observed for PSII and PSI could be the result of different regulation pathways. Because, it was previously demonstrated that oxygenic photosynthesis produces various active oxygen species with harmful effects on PSII and that protein D1 is turned over quickly in cells under light conditions, we could hypothesize that the down-regulated protein ratio of PSII under light/dark cycle is the result of an excessive turnover occurring at the end of the day when compared to the dark phase [42]. Moreover, it was recently reported in the cyanobacterium *Cyanothece* sp. Strain ATCC 51142 that genes encoding for PSII reaction center proteins were transcribed and also translated in the dark phase [21]. Those proteins translated during the dark phase and not being subjected to the same light-dependent turnover could thus explain a higher protein content in the dark phase relative to the light phase in LD-grown cultures of *A. platensis*. Furthermore, a unifying hypothesis for the protein fold changes of PSII and PSI as well as cytochrome f (ARTHROv3_170006) and ATP synthase (ARTHROv3_630063; ARTHROv3_630062) would be that *A. platensis* anticipates the future the following day by synthesizing the needed photosynthesis-related proteins at the light/dark transition phase. The carbon fixation would preferentially occur during the dark phase manifested in the changes of protein abundance with a higher content of three Calvin-cycle associated-proteins during the dark phase (ARTHROv3_190004, L/D ratio of 0.5; ARTHROv3_810066 and ARTHROv3_810107, L/D ratio of 0.7) (Table 1).

In contrast, proteins of the phycobilisome (ARTHROv3_1100012; ARTHROv3_5910003; ARTHROv3_1100014; ARTHROv3_1280008), which play a major role in the transfer of light energy to chlorophyll of PSII, were found to be more abundant (from 1.2- to 1.5-fold) during the light phase than during the dark period. This result was confirmed by pigment analysis (Figure 6). Indeed, the pigment of phycocyanin was found to be more abundant during the end of the day compared to the end of the dark phase (Figure 6), confirming our proteomics results showing a ratio >1.25 (Table 1). *A. platensis* has no phycoerythrin and the phycobilisome is composed exclusively of allophycocyanin and phycocyanin [1]. It is not surprising that phycobilisome pigments involved in the channeling of solar energy are over-represented during the sunlight phase compared to the dark phase. Phycobilisome synthesis would be light-dependent and not regulated by a specific clock. The expression of the most abundant proteins in *A. platensis*, the allophycocyanins and phycocyanins, also well known as efficient antioxidants, could be more affected by the availability of light than by a circadian cycle. Further experiments are necessary to confirm whether phycocyanin expression follows the same periodicity in a constant light environment and determine whether regulation occurs according to an internal rhythm, or according to a light dependent rmechanism. Our proteomics results also highlight the importance of translational-related proteins in the light phase compared to the dark phase, with the cellular increase of 4 ribosomal proteins. The identification of two proteins annotated as hypothetical (ARTHROv3_1350003; ARTHROv3_680013) and determined to be controlled rhythmically raises the question of their function under light/dark cultivation (Table 1). Among the 30 significant differentially expressed proteins, we could identify only one protein whose expression level changed more than threefold throughout the circadian cycle. This protein was identified as a glycine-rich RNA binding protein, rbp-like protein (ARTHROv3_870040). Genes encoding glycine-rich proteins were determined to be four-fold upregulated at the transcriptional level in cold-stressed plants of *Arabidopsis thaliana*, and they might be subjected to light/dark mediated regulation [42]–[44]. A transcript encoding a glycine-rich RNA binding protein in *A. thaliana* was found to undergo circadian oscillation with peaks levels at the end of the day [44], thus confirming our result with an L/D ratio >3. There is increasing knowledge regarding the functional roles of glycine-rich RNA binding proteins (GRPs) in higher plants compared to cyanobacteria. These proteins are composed of an RNA-recognition motif at the N-terminus of the protein and a glycine-rich region at the C-terminus, and for this reason they are referred to as the glycine-rich RNA binding proteins. The expression levels of some RNA-binding protein genes (rbp) were found to be up-regulated in the cyanobacterium *Synechocystis* when they were stressed by low temperature and light [45]. RNA binding proteins are known to accumulate following cold stress and play key roles in regulating transcription termination. Interestingly, it was previously demonstrated that ribonucleoprotein complexes played a role in the post-transcriptional regulation of circadian clocks [46]. Quantified proteins are likely to represent the most abundant ones and further experiments are necessary to confirm this preliminary quantitative proteomics study.

Importantly, it has been demonstrated that cyanobacteria that contain the *kaiA* gene maintain periodic expression under constant light conditions. However, *Prochlorococcus* does not because it does not harbour the *kaiA* gene [39], [47]. Note that to confirm if our present results are correlated well with an endogenous clock in *A. platensis*, the observed patterns might persist in constant light once the population has been entrained on a LD cycle. This possibility requires further experimental investigation to conclude on a circadian clock or on a light dependent regulation. It could be highly valuable to sample more extensively both light and dark phases to more accurately describe the peaks of protein regulation.

*A. platensis* is able to sense and react to the LD cycle, a process based on the diurnal cycle on Earth that does not exist during space flight. Interestingly, the photoaccumulation phenomenon observed in *C. reinhardtii*, which follows a circadian rhythm was found to persist in outer space, aboard a spacecraft, when the cells orbited the earth every 90 min [48]. The rhythms continued for at least 6 days in microgravity without any terrestrial clue of the time of day. Therefore, these studies of *C. reinhardtii* showed that the circadian clock can run independently of daily cycles of gravity, magnetism, and cosmic ray irradiation, which is a key point for the MELiSSA project. It was also previously reported that the sensitivity of *C. reinhardtii* towards the harmful UV radiation was modulated according the LD cycle, exhibiting increased UV-resistance at the beginning of the day and the end of the night [49]. From those two reported results, multiple stress resistance studies (microgravity, cosmic ray irradiation) in either LD cycles or constant light should provide valuable data for future perspectives to select which growth condition would be more suitable for maintaining a good bioreactor performance of *A. platensis* in long-term manned missions; thus, the data represent a key milestone for bioreactor design. Taken together, additional space experiments in continuous bioreactors are crucial to draw final conclusions concerning the space flight impact for MELiSSA. New space experiments need to be conducted under conditions mimicking the future MELiSSA loop conditions (with continuous light or a LD cycle), and should include a detailed nutritive analysis in addition to proteomic profiling to determine the optimum condition for supplying food to space crews.

## Supporting Information

Table S1 **Spreadsheet presenting the complete list of the 1306 proteins identified by Mascot [Excel File].**
(XLS)

Table S2 **List of proteins only identified within the flowthrough fraction of the Cu-IMAC experiment (106 proteins).**
(PDF)

Table S3 **List of proteins only identified within the elution faction of the Cu-IMAC experiment (558 proteins).**
(PDF)

Table S4 **List of proteins identified in the flowthrough and elution factions of the Cu-IMAC experiment (107 proteins).**
(PDF)

Data S1 **This file contains 12 sheets with the identification data provided by Mascot and quantification (ICPL rep1 and ICPL rep2) provided by Mascot Distiller [Excel file].**
(ZIP)
