# Supplementary material for: Proteome-Wide Analysis and Diel Proteomic Profiling of the Cyanobacterium Arthrospira platensis PCC 8005
Source: PLoS One. 2014 Jun 10;9(6):e99076. doi: 10.1371/journal.pone.0099076 (PMC4051694; doi:10.1371/journal.pone.0099076)
Supplement: Table S2 — List of proteins only identified within the flowthrough fraction of the Cu-IMAC experiment (106 proteins). (PDF) [file pone.0099076.s002.pdf]

Table S2. List of identified proteins only identified within the flowthrough fraction of the Cu-IMAC experiment (106 proteins)

Accession Number	Name of proteins	empAI values
ARTHROv3_1400045	trxA1 Thioredoxin-1	15.37
ARTHROv3_210030	psbU Photosystem II 12 kDa extrinsic protein precursor (PS II complex 12 kDa extrinsic protein)	4.65
ARTHROv3_1500008	rbp1 Glycine-rich RNA-binding protein. rbp-like	4.4
ARTHROv3_960038	psb28 Photosystem II reaction center psb28 protein (W protein) (Photosystem II 13 kDa protein)	4.35
ARTHROv3_610004	exported protein of unknown function	4.18
ARTHROv3_1130127	conserved protein of unknown function	3.85
ARTHROv3_1160015	putative 30S ribosomal protein PSRP-3	3.79
ARTHROv3_930037	rpsM 30S ribosomal subunit protein S13	3.5
ARTHROv3_1530022	frr ribosome recycling factor	3.25
ARTHROv3_920007	gcvH Glycine cleavage system H protein	2.67
ARTHROv3_3580002	conserved hypothetical protein	2.43
ARTHROv3_1130026	pspA2 phage shock protein A. PspA	2.42
ARTHROv3_810042	conserved hypothetical protein	2.04
ARTHROv3_1210003	putative Peptidyl-prolyl cis-trans isomerase. cyclophilin family	1.78
ARTHROv3_960012	putative Peptidyl-prolyl cis-trans isomerase. cyclophilin family	1.75
ARTHROv3_1160019	putative two-component response regulator	1.74
ARTHROv3_1580005	anti-sigma factor antagonist	1.67
ARTHROv3_6530002	conserved protein of unknown function	1.61
ARTHROv3_1130066	fbp peptidyl-prolyl cis-trans isomerase	1.49
ARTHROv3_960021	fdx Ferredoxin (2Fe-2S)	1.25
ARTHROv3_760043	conserved hypothetical protein	1.23
ARTHROv3_1550089	conserved hypothetical protein	1.16
ARTHROv3_490005	kaiB Circadian clock protein KaiB	1.16
ARTHROv3_1130017	conserved hypothetical protein (secreted)	1.12
ARTHROv3_1380014	conserved hypothetical protein	1.09
ARTHROv3_280005	hypothetical protein	1.09
ARTHROv3_130017	hypothetical protein	1.01
ARTHROv3_840052	conserved hypothetical protein	1.01
ARTHROv3_320015	conserved hypothetical protein	0.96
ARTHROv3_680018	conserved hypothetical protein	0.87
ARTHROv3_4410004	conserved protein of unknown function	0.76
ARTHROv3_590034	cchB putative carboxysome-like ethanolaminosome structural protein	0.73
ARTHROv3_1420020	pedR PedR transcriptional regulator. LuxR family	0.67
ARTHROv3_1730001	conserved hypothetical protein	0.66
ARTHROv3_1090002	rbp4 Glycine-rich RNA-binding protein. rbp-like	0.64
ARTHROv3_890005	cheW3 CheW chemotaxis protein	0.61
ARTHROv3_1130133	hypothetical protein	0.6
ARTHROv3_120009	conserved protein of unknown function	0.55
ARTHROv3_690106	conserved hypothetical protein	0.54
ARTHROv3_1130042	conserved hypothetical protein	0.51
ARTHROv3_1410008	conserved hypothetical protein	0.51
ARTHROv3_480007	conserved hypothetical protein (fragment)	0.49
ARTHROv3_650002	conserved protein of unknown function	0.48
ARTHROv3_10049	hypothetical protein	0.46
ARTHROv3_150014	conserved hypothetical protein	0.45
ARTHROv3_200040	conserved protein of unknown function	0.43
ARTHROv3_10084	conserved protein of unknown function	0.41
ARTHROv3_200027	hypothetical protein	0.4
ARTHROv3_1050033	conserved protein of unknown function	0.39
ARTHROv3_1420003	conserved hypothetical protein	0.39
ARTHROv3_830023	conserved hypothetical protein	0.39
ARTHROv3_1530023	chlP geranylgeranyl reductase	0.38
ARTHROv3_960008	conserved hypothetical protein (secreted)	0.32
ARTHROv3_370010	rbp3 Glycine-rich RNA-binding protein. rbp-like	0.31
ARTHROv3_460008	petF Ferredoxin (2Fe-2S)	0.31
ARTHROv3_630008	conserved hypothetical protein	0.29
ARTHROv3_1130075	conserved hypothetical protein	0.27
ARTHROv3_1420069	trxA2 Thioredoxin-1	0.27
ARTHROv3_420034	hypothetical protein	0.27
ARTHROv3_750010	putative haemolysin-type calcium-binding toxin. RTX-like (expressed)	0.27
ARTHROv3_860013	CheY chemotaxis response regulator	0.26
ARTHROv3_1030003	conserved hypothetical protein (secreted)	0.24
ARTHROv3_1210011	atpC ATP synthase epsilon chain	0.23
ARTHROv3_150048	conserved hypothetical protein	0.23
ARTHROv3_1470006	conserved protein of unknown function	0.21
ARTHROv3_2180001	cynS cyanase	0.21
ARTHROv3_750038	conserved hypothetical protein	0.21

ARTHROv3_10134	conserved hypothetical protein (secreted)	0.19
ARTHROv3_750043	conserved hypothetical protein (excreted)	0.19
ARTHROv3_1240009	conserved hypothetical protein (secreted)	0.18
ARTHROv3_550002	conserved hypothetical protein (fragment)	0.17
ARTHROv3_590007	pyrR Bifunctional protein pyrR	0.17
ARTHROv3_230008	infC translation initiation factor IF-3	0.16
ARTHROv3_1300010	tyrS tyrosyl-tRNA synthetase	0.15
ARTHROv3_1530086	N-acetylmuramoyl-L-alanine amidase	0.15
ARTHROv3_570048	avtA valine-pyruvate aminotransferase	0.14
ARTHROv3_840015	rpiA Ribose-5-phosphate isomerase A	0.13
ARTHROv3_10053	putative [Myosin heavy-chain] kinase	0.12
ARTHROv3_1080002	putative D-alanyl-D-alanine dipeptidase	0.12
ARTHROv3_1130022	conserved hypothetical protein	0.12
ARTHROv3_1420068	fabG1 3-oxoacyl-[acyl-carrier-protein] reductase	0.12
ARTHROv3_420027	conserved hypothetical protein (membrane)	0.12
ARTHROv3_560008	conserved protein of unknown function	0.12
ARTHROv3_690033	Efflux transporter. RND/HAE family. Membrane Fusion Protein subunit	0.12
ARTHROv3_810063	putative estradiol 17-beta-dehydrogenase	0.12
ARTHROv3_1170018	conserved hypothetical protein	0.11
ARTHROv3_1490004	conserved hypothetical protein	0.11
ARTHROv3_10050	hypothetical protein	0.1
ARTHROv3_10052	hypothetical protein	0.1
ARTHROv3_1470003	conserved hypothetical protein	0.1
ARTHROv3_1130084	fmt Methionyl-tRNA formyltransferase	0.09
ARTHROv3_1550081	mreB cell wall structural complex MreBCD. actin-like component MreB	0.09
ARTHROv3_200038	adh alcohol desydrogenase. zinc binding.	0.09
ARTHROv3_750033	cbpA curved DNA-binding protein. DnaJ homologue that functions as a co-chaperone of DnaK	0.09
ARTHROv3_10011	transposase. IS605 family. OrfB	0.08
ARTHROv3_1130057	hypothetical protein	0.08
ARTHROv3_750031	Peptidase S1 and S6. chymotrypsin/Hap	0.08
ARTHROv3_1450082	intB1 site-specific recombinase	0.06
ARTHROv3_1200003	Hemolysin-type calcium-binding region (fragment)	0.05
ARTHROv3_2100001	protease. ATP-dependent zinc-metallo (fragment)	0.05
ARTHROv3_400007	ATP-binding protein of ABC transporter	0.05
ARTHROv3_1490058	dnaK2 Chaperone protein DnaK	0.04
ARTHROv3_560002	general secretion pathway protein D	0.04
ARTHROv3_570026	serine/threonine kinase	0.04
ARTHROv3_1150004	mutS DNA methyl-directed mismatch repair protein MutS	0.03
ARTHROv3_1550001	protein of unknown function	0.01
