# Supplementary material for: Proteome-Wide Analysis and Diel Proteomic Profiling of the Cyanobacterium Arthrospira platensis PCC 8005
Source: PLoS One. 2014 Jun 10;9(6):e99076. doi: 10.1371/journal.pone.0099076 (PMC4051694; doi:10.1371/journal.pone.0099076)
Supplement: Table S3 — List of proteins only identified within the elution faction of the Cu-IMAC experiment (558 proteins). (PDF) [file pone.0099076.s003.pdf]

Table S3. List of identified proteins from the elution faction of the Cu-IMAC experiment (558 proteins)

Accession Number	Name of proteins	empAI values
ARTHROv3_6030002	bcp2 Bacterioferritin comigratory protein	6.37
ARTHROv3_850006	glpX D-fructose 1.6-bisphosphatase class II	6
ARTHROv3_870040	rbp2 Glycine-rich RNA-binding protein. rbp-like	5.65
ARTHROv3_420090	cbbS Ribulose bisphosphate carboxylase small chain (RuBisCO small subunit)	5.31
ARTHROv3_840073	conserved protein of unknown function	4.77
ARTHROv3_1400078	psaD Photosystem I reaction center subunit II (Photosystem I 16 kDa polypeptide) (PSI-D)	4.71
ARTHROv3_190013	conserved hypothetical protein	4.67
ARTHROv3_330005	petH Ferredoxin--NADP reductase (FNR)	4.39
ARTHROv3_150049	cpcG phycobilisome rod-core linker protein	3.99
ARTHROv3_810109	Ferredoxin thioredoxin reductase alpha chain	3.85
ARTHROv3_930039	adk Adenylate kinase (ATP-AMP transphosphorylase)	3.81
ARTHROv3_3290003	conserved hypothetical protein	3.58
ARTHROv3_730028	ndk nucleoside diphosphate kinase	3.44
ARTHROv3_680027	conserved hypothetical protein	3.37
ARTHROv3_1380022	conserved protein of unknown function	3.27
ARTHROv3_1430015	pphA PP2C/PPM-type Ser/thr protein phosphatase	3.05
ARTHROv3_1530020	alkyl hydroperoxide reductase/ thiol specific antioxidant/ Mal allergen	3
ARTHROv3_6530003	conserved protein of unknown function	2.57
ARTHROv3_1620011	psaC Photosystem I iron-sulfur center (Photosystem I subunit VII) (9 kDa polypeptide) (PSI-C) (PsaC)	2.56
ARTHROv3_690124	conserved protein of unknown function	2.47
ARTHROv3_470002	conserved hypothetical protein	2.34
ARTHROv3_720009	conserved hypothetical protein	2.34
ARTHROv3_740074	rbfA 30s ribosome binding factor A	2.27
ARTHROv3_1340001	conserved hypothetical protein	2.25
ARTHROv3_690123	conserved hypothetical protein	2.22
ARTHROv3_4470001	conserved protein of unknown function	2.15
ARTHROv3_1090007	hup2 Histone-like bacterial DNA-binding protein. HU-like	2.11
ARTHROv3_1400067	ureA Urease subunit gamma (Urea amidohydrolase subunit gamma)	2.01
ARTHROv3_400031	conserved hypothetical protein	2.01
ARTHROv3_810118	conserved protein of unknown function	1.91
ARTHROv3_2840002	conserved hypothetical protein	1.89
ARTHROv3_1300027	pgk phosphoglycerate kinase	1.87
ARTHROv3_430028	gor glutathione oxidoreductase	1.86
ARTHROv3_300024	serine protease. S1C family	1.82
ARTHROv3_1110010	conserved hypothetical protein	1.77
ARTHROv3_310029	conserved hypothetical protein	1.74
ARTHROv3_1510054	pentapeptide repeat-containing protein	1.72
ARTHROv3_1460003	mdh malate dehydrogenase	1.69
ARTHROv3_1400120	transcriptional regulator. Crp-like	1.64
ARTHROv3_1130065	conserved hypothetical protein	1.59
ARTHROv3_1550032	conserved hypothetical protein	1.59
ARTHROv3_1540029	putative Subtilisin-like serine protease	1.58
ARTHROv3_1520003	hisB Imidazoleglycerol-phosphate dehydratase	1.55
ARTHROv3_810100	pgi Glucose-6-phosphate isomerase	1.51
ARTHROv3_3700001	glgC Glucose-1-phosphate adenylyltransferase	1.48
ARTHROv3_660038	terX tellurium resistance protein	1.46
ARTHROv3_730012	ccmk2 microcompartments protein	1.43
ARTHROv3_1130077	Putative pterin-4-alpha-carbinolamine dehydratase	1.42
ARTHROv3_660039	terD tellurium resistance protein	1.41
ARTHROv3_550016	ssb Single-stranded DNA-binding protein (fragment)	1.4
ARTHROv3_630076	conserved protein of unknown function	1.36
ARTHROv3_1050054	ribH 6.7-dimethyl-8-ribityllumazine synthase	1.35
ARTHROv3_690135	ftrC ferredoxin--thioredoxin reductase catalytic chain	1.35
ARTHROv3_1000010	putative phycobilisome protein	1.33
ARTHROv3_1280009	glnA glutamine synthetase	1.32
ARTHROv3_430054	conserved hypothetical protein	1.3
ARTHROv3_90006	conserved hypothetical protein (fragment)	1.28
ARTHROv3_590032	cchA putative carboxysome-like ethanolaminosome structural protein. ethanolamine utilization protein	1.27
ARTHROv3_930035	rpoA RNA polymerase. alpha subunit	1.27
ARTHROv3_620013	dapA Dihydrodipicolinate synthase (DHDPS)	1.25
ARTHROv3_370022	conserved hypothetical protein	1.23
ARTHROv3_750037	msrB methionine sulfoxide reductase B	1.19
ARTHROv3_1050056	nblB2 Phycocyanin alpha phycocyanobilin lyase related protein	1.17
ARTHROv3_630062	atpH ATP synthase delta chain; ATP synthase F1. delta subunit	1.17
ARTHROv3_1400046	guaB inositol-5-monophosphate dehydrogenase	1.16
ARTHROv3_810113	ferredoxin-like	1.16
ARTHROv3_1490011	yccU CoA-binding protein	1.14
ARTHROv3_750045	cysC Adenylyl-sulfate kinase	1.12
ARTHROv3_4000003	putative FdxN element excision controlling factor protein. XisI-like	1.09
ARTHROv3_4780002	nusG Transcription antitermination protein NusG	1.09
ARTHROv3_1610013	conserved hypothetical protein	1.08
ARTHROv3_1460004	conserved hypothetical protein	1.06
ARTHROv3_2380001	sir fragment of sulfite reductase subunit beta (part 2)	1.06
ARTHROv3_420013	glnB protein P-II	1.06
ARTHROv3_6840001	hinT purine nucleoside phosphoramidase	1.05

ARTHROv3_310059	conserved hypothetical protein	1.03
ARTHROv3_1070005	talB transaldolase B	1.02
ARTHROv3_790008	narM nitrate reductase accessory protein	1.01
ARTHROv3_1110021	putative carboxymethylenebutenolidase	1
ARTHROv3_430022	conserved exported protein of unknown function	1
ARTHROv3_6740003	rpsA1 30S ribosomal protein S1	0.99
ARTHROv3_960002	conserved hypothetical protein	0.99
ARTHROv3_1000007	fdx2 Ferredoxin (2Fe-2S)	0.98
ARTHROv3_740050	conserved hypothetical protein	0.97
ARTHROv3_740062	conserved hypothetical protein	0.97
ARTHROv3_10117	putative Anti-sigma factor antagonist (fragment)	0.96
ARTHROv3_400034	hypothetical protein	0.95
ARTHROv3_420020	putative Molybdopterin biosynthesis protein. MoeB-like	0.92
ARTHROv3_930069	hypothetical protein	0.92
ARTHROv3_360001	conserved hypothetical protein	0.9
ARTHROv3_660026	sat Sulfate adenylyltransferase	0.9
ARTHROv3_3200002	putative Phosphoglycerate mutase. GpmB-like	0.89
ARTHROv3_1020008	putative branched-chain amino acid aminotransferase protein (BCAT); IlvE like protein	0.88
ARTHROv3_1500010	murF UDP-N-acetylmuramoylalanyl-D-glutamyl-2. 6-diaminopimelate--D-alanyl-D-alanine ligase	0.88
ARTHROv3_690036	malQ 4-alpha-glucanotransferase	0.88
ARTHROv3_2770002	conserved hypothetical protein	0.87
ARTHROv3_300032	ycf3 Photosystem I assembly protein Ycf3	0.86
ARTHROv3_690061	conserved hypothetical protein	0.86
ARTHROv3_10093	tpiA triosephosphate isomerase	0.85
ARTHROv3_1100012	cpcC1 Phycobilisome 32 kDa linker polypeptide. phycocyanin-associated. rod 1	0.85
ARTHROv3_1140025	putative response regulator. SirA-like	0.84
ARTHROv3_390014	conserved hypothetical protein	0.84
ARTHROv3_900013	conserved hypothetical protein	0.84
ARTHROv3_1050043	hisI Phosphoribosyl-AMP cyclohydrolase	0.83
ARTHROv3_1320002	conserved protein of unknown function	0.83
ARTHROv3_1490016	grxC1 glutaredoxin-3. GrxC-like	0.83
ARTHROv3_570046	SH3 type 3 domain protein	0.81
ARTHROv3_840087	Universal stress protein. UspA-like	0.79
ARTHROv3_5300008	protein of unknown function	0.78
ARTHROv3_130043	conserved hypothetical protein	0.77
ARTHROv3_2810001	1.4-alpha-glucan branching enzyme (fragment)	0.77
ARTHROv3_4330001	Two component transcriptional regulator. winged helix family	0.77
ARTHROv3_790052	conserved protein of unknown function	0.77
ARTHROv3_1550087	putative Arsenite-transporting ATPase	0.76
ARTHROv3_210023	leuB 3-isopropylmalate dehydrogenase	0.76
ARTHROv3_400038	conserved hypothetical protein	0.75
ARTHROv3_810105	conserved protein of unknown function	0.75
ARTHROv3_810062	conserved hypothetical protein (secreted)	0.74
ARTHROv3_1240014	putative Peptidyl-prolyl cis-trans isomerase. PpiC-like	0.73
ARTHROv3_670003	conserved protein of unknown function	0.73
ARTHROv3_690019	ahcY Adenosylhomocysteinase	0.73
ARTHROv3_120030	hisH Imidazole glycerol phosphate synthase subunit hisH	0.71
ARTHROv3_2150002	ispF 2C-methyl-D-erythritol 2.4-cyclodiphosphate synthase	0.7
ARTHROv3_430056	atoB acetyl-CoA acetyltransferase	0.7
ARTHROv3_1050057	conserved hypothetical protein	0.69
ARTHROv3_1490041	por Chlorophyll synthase / NADPH-protochlorophyllide oxidoreductase	0.69
ARTHROv3_2170002	3-beta hydroxysteroid dehydrogenase/isomerase	0.68
ARTHROv3_370020	hypothetical protein	0.66
ARTHROv3_590033	ccmK Carbon dioxide-concentrating mechanism protein	0.65
ARTHROv3_810120	putative Short-chain dehydrogenase/reductase family (SDR); Glucose/ribitol dehydrogenase.	0.65
ARTHROv3_1260011	argI ornithine carbamoyltransferase 1	0.64
ARTHROv3_1420022	rfpX fluorescence recovery protein (RFP)	0.64
ARTHROv3_1530067	rpe D-ribulose-5-phosphate 3-epimerase	0.64
ARTHROv3_240046	conserved protein of unknown function	0.64
ARTHROv3_4350002	periplasmic mercuric ion binding protein	0.63
ARTHROv3_1100006	ssb Single-stranded DNA-binding protein	0.62
ARTHROv3_1620071	conserved hypothetical protein	0.62
ARTHROv3_690011	conserved hypothetical protein	0.62
ARTHROv3_840047	sixA Phosphohistidine phosphatase. SixA	0.62
ARTHROv3_1550004	putative DegT/DnrJ/EryC1/StrS aminotransferase protein family	0.61
ARTHROv3_160028	sqr Sulfide:quinone oxidoreductase	0.61
ARTHROv3_20004	conserved hypothetical protein (secreted)	0.6
ARTHROv3_10029	conserved hypothetical protein	0.58
ARTHROv3_1130036	Putative thylakoid formation protein. Thf1-like	0.58
ARTHROv3_870023	conserved hypothetical protein	0.58
ARTHROv3_1130038	anti-sigma factor antagonist	0.57
ARTHROv3_1510075	fabI Enoyl-[acyl-carrier-protein] reductase [NADH]	0.57
ARTHROv3_2560002	rmlC dTDP-4-deoxyrhamnose-3.5-epimerase	0.57
ARTHROv3_430060	pnp polynucleotide phosphorylase/polyadenylase	0.57
ARTHROv3_910015	cphA Cyanophycin synthetase	0.56
ARTHROv3_110003	putative phosphoketolase	0.55
ARTHROv3_1530015	lpdA Dihydrolipoyl dehydrogenase	0.55
ARTHROv3_1050014	ureG Urease accessory protein UreG	0.54

ARTHROv3_680003	hypothetical protein	0.54
ARTHROv3_830002	ilvD Dihydroxy-acid dehydratase	0.54
ARTHROv3_870050	suhB inositol monophosphatase	0.54
ARTHROv3_1540031	putative L-PSP (mRNA) endoribonuclease (putative translation initiation inhibitor. YjgF-like)	0.53
ARTHROv3_730015	asnS asparaginyl tRNA synthetase	0.53
ARTHROv3_830003	conserved hypothetical protein	0.53
ARTHROv3_850024	dapB Dihydrodipicolinate reductase	0.53
ARTHROv3_1530039	conserved hypothetical protein	0.52
ARTHROv3_160003	clpP3 proteolytic subunit of ClpA-ClpP and ClpX-ClpP ATP-dependent serine proteases	0.52
ARTHROv3_660024	conserved exported protein of unknown function	0.52
ARTHROv3_380032	acsA Acetyl-coenzyme A synthetase	0.51
ARTHROv3_1160005	Universal stress protein. UspA-like	0.5
ARTHROv3_1380053	putative Aldo/keto reductase	0.5
ARTHROv3_1620074	putative Aldo/keto reductase	0.5
ARTHROv3_1380003	mtaP S-methyl-5'-thioadenosine phosphorylase	0.49
ARTHROv3_240024	aroQ 3-dehydroquinate dehydratase type 2	0.48
ARTHROv3_430050	Glycine oxidase ThiO	0.48
ARTHROv3_690134	conserved hypothetical protein	0.48
ARTHROv3_960036	putative serine--pyruvate aminotransferase	0.48
ARTHROv3_1060001	conserved hypothetical protein	0.46
ARTHROv3_1380031	fmdA formamidase (formamide amidohydrolase)	0.46
ARTHROv3_1530103	conserved protein of unknown function	0.46
ARTHROv3_1540002	conserved hypothetical protein	0.46
ARTHROv3_220018	devR two-component response regulator	0.46
ARTHROv3_380030	putative structural maintenance of chromosomes (SMC) protein	0.46
ARTHROv3_10138	conserved hypothetical protein	0.45
ARTHROv3_1030004	putative transketolase	0.45
ARTHROv3_1140005	upp Uracil phosphoribosyltransferase	0.45
ARTHROv3_1520025	conserved hypothetical protein	0.45
ARTHROv3_3150001	NUDIX hydrolase (fragment)	0.45
ARTHROv3_420045	conserved protein of unknown function	0.45
ARTHROv3_750035	hspA heat shock protein A	0.45
ARTHROv3_1180012	conserved hypothetical protein	0.44
ARTHROv3_1280013	conserved hypothetical protein	0.44
ARTHROv3_1450032	Ankyrin repeat protein (fragment?)	0.44
ARTHROv3_1470023	conserved hypothetical protein	0.44
ARTHROv3_1490056	thiS Thiamine biosynthesis protein	0.44
ARTHROv3_6070001	conserved hypothetical protein	0.44
ARTHROv3_130044	conserved hypothetical protein	0.43
ARTHROv3_1440052	conserved hypothetical protein	0.43
ARTHROv3_210031	polyketide cyclase/dehydrase	0.43
ARTHROv3_760029	putative ferredoxin (fragment)	0.43
ARTHROv3_770018	arsA arsenite-activated ATPase ArsA	0.43
ARTHROv3_120021	putative Phosphoglycolate phosphatase	0.42
ARTHROv3_1620052	conserved hypothetical protein	0.42
ARTHROv3_1880004	conserved exported protein of unknown function	0.42
ARTHROv3_340020	xfp D-xylulose 5-phosphate/D-fructose 6-phosphate phosphoketolase	0.42
ARTHROv3_690060	conserved hypothetical protein	0.42
ARTHROv3_750015	conserved hypothetical protein	0.42
ARTHROv3_1090011	conserved hypothetical protein	0.41
ARTHROv3_1420039	conserved hypothetical protein	0.41
ARTHROv3_1540040	trpB Tryptophan synthase beta chain	0.41
ARTHROv3_870052	hisZ ATP phosphoribosyltransferase regulatory subunit	0.41
ARTHROv3_930038	infA translation initiation factor IF-1	0.41
ARTHROv3_1430009	pfkA1 6-phosphofructokinase I	0.4
ARTHROv3_1530063	conserved hypothetical protein (secreted)	0.4
ARTHROv3_120045	putative Subtilisin-like serine protease. PatA-like	0.39
ARTHROv3_5610002	protein of unknown function	0.39
ARTHROv3_1130025	conserved hypothetical protein	0.38
ARTHROv3_1350003	hypothetical protein	0.38
ARTHROv3_1410011	surE broad specificity 5'(3')-nucleotidase and polyphosphatase	0.38
ARTHROv3_1550090	conserved hypothetical protein	0.38
ARTHROv3_170007	petC Cytochrome b6-f complex iron-sulfur subunit 1 (Rieske iron-sulfur protein 1)	0.38
ARTHROv3_2670001	conserved exported protein of unknown function	0.38
ARTHROv3_300003	glgA Glycogen synthase 1	0.38
ARTHROv3_320022	hisA 5-phosphoribosylaminomethylideneamino imidazole-4-carboxamide isomerase	0.38
ARTHROv3_690009	conserved protein of unknown function	0.38
ARTHROv3_690043	ychF translation-associated GTPase	0.38
ARTHROv3_3340003	fbp fructose-1.6-bisphosphatase I	0.37
ARTHROv3_760021	glgX1 glycogen debranching enzyme	0.37
ARTHROv3_150023	conserved hypothetical protein	0.36
ARTHROv3_310071	bcp3 Bacterioferritin comigratory protein	0.36
ARTHROv3_730021	conserved hypothetical protein	0.36
ARTHROv3_810093	conserved hypothetical protein	0.36
ARTHROv3_1130101	conserved hypothetical protein	0.35
ARTHROv3_120038	dfa putative diflavin flavoprotein A 3	0.35
ARTHROv3_1400127	Inorganic pyrophosphatase	0.35
ARTHROv3_310027	hoxY NAD-reducing hydrogenase. small subunit	0.35

ARTHROv3_810090	conserved protein of unknown function	0.35
ARTHROv3_1020007	conserved hypothetical protein	0.34
ARTHROv3_1050044	cysS cysteinyl-tRNA synthetase	0.34
ARTHROv3_1130109	conserved protein of unknown function	0.34
ARTHROv3_3140002	maltooligosyltrehalose synthase (fragment)	0.34
ARTHROv3_780001	conserved protein of unknown function	0.34
ARTHROv3_810069	conserved hypothetical protein	0.34
ARTHROv3_1400080	phzF Phenazine biosynthesis protein PhzF family	0.33
ARTHROv3_310070	conserved protein of unknown function	0.33
ARTHROv3_570002	aspS aspartyl-tRNA synthetase	0.33
ARTHROv3_630082	conserved protein of unknown function	0.33
ARTHROv3_1440069	PP2C/PPM-type Ser/Thr protein phosphatase	0.32
ARTHROv3_1520007	carbohydrate kinase. YjeF related protein	0.32
ARTHROv3_1530055	ndhH NAD(P)H-quinone oxidoreductase chain H	0.32
ARTHROv3_1550023	putative plasmid stabilization system protein	0.32
ARTHROv3_240017	conserved hypothetical protein	0.32
ARTHROv3_3140001	putative maltooligosyltrehalose trehalohydrolase (fragment)	0.32
ARTHROv3_380010	conserved hypothetical protein	0.32
ARTHROv3_690041	cobL precorrin-6B methylase	0.32
ARTHROv3_690110	conserved hypothetical protein	0.32
ARTHROv3_840111	purS Phosphoribosylformylglycinamidine synthase. PurS subunit	0.32
ARTHROv3_850022	cobH2 Precorrin-8X methylmutase	0.32
ARTHROv3_1130081	clpS2 ATP-dependent Clp protease adapter protein ClpS	0.31
ARTHROv3_1140004	conserved hypothetical protein	0.31
ARTHROv3_1400114	putative plasmid stabilization system protein	0.31
ARTHROv3_1440031	conserved hypothetical protein	0.31
ARTHROv3_370003	aroA 3-phosphoshikimate 1-carboxyvinyltransferase	0.31
ARTHROv3_1130044	conserved hypothetical protein	0.3
ARTHROv3_120010	putative UDP-glucuronate decarboxylase	0.3
ARTHROv3_1320010	glgP glycogen phosphorylase; Glycogen/starch/alpha-glucan phosphorylases	0.3
ARTHROv3_1490017	gshB glutathione synthetase	0.3
ARTHROv3_240037	dihydroorotase. multifunctional complex type	0.3
ARTHROv3_240048	putative Succinate-semialdehyde dehydrogenase (NAD(P)(+)). GabD-like	0.3
ARTHROv3_840093	rpoC2 DNA-directed RNA polymerase subunit beta'	0.3
ARTHROv3_930059	putative antibiotic biosynthesis monooxygenase	0.3
ARTHROv3_1610011	putative pfkB family carbohydrate kinase; Adenosine kinase	0.29
ARTHROv3_300040	conserved hypothetical protein	0.29
ARTHROv3_1240007	gvpJ Gas vesicle synthesis protein GvpJ	0.28
ARTHROv3_1420009	Peptidase C14. caspase catalytic subunit p20	0.28
ARTHROv3_160005	clpP1 proteolytic subunit of ClpA-ClpP and ClpX-ClpP ATP-dependent serine proteases	0.28
ARTHROv3_1920001	clpP proteolytic subunit of ClpA-ClpP and ClpX-ClpP ATP-dependent serine proteases	0.28
ARTHROv3_680026	grx monothiol glutaredoxin	0.28
ARTHROv3_810092	Conserved protein of unknown function; putative cupin region	0.28
ARTHROv3_1000009	conserved hypothetical protein	0.27
ARTHROv3_1010004	putative hydroxyacylglutathione hydrolase	0.27
ARTHROv3_1170019	gltX Glutamyl-tRNA synthetase	0.27
ARTHROv3_1470026	conserved hypothetical protein	0.27
ARTHROv3_150077	aroC chorismate synthase	0.27
ARTHROv3_1530012	FAD dependent oxidoreductase	0.27
ARTHROv3_1610019	conserved hypothetical protein	0.27
ARTHROv3_280003	conserved hypothetical protein	0.27
ARTHROv3_640017	hemE Uroporphyrinogen decarboxylase	0.27
ARTHROv3_680023	putative Subtilisin-like serine protease	0.27
ARTHROv3_910011	trmD tRNA (guanine-N(1)-)-methyltransferase (M1G- methyltransferase) (tRNA	0.27
ARTHROv3_1110001	conserved protein of unknown function	0.26
ARTHROv3_1430044	gvpV Gas vesicle protein GvpV	0.26
ARTHROv3_1440044	adhE fused acetaldehyde-CoA dehydrogenase; iron-dependent alcohol dehydrogenase	0.26
ARTHROv3_1610035	clpS1 ATP-dependent Clp protease adapter protein ClpS	0.26
ARTHROv3_310057	conserved hypothetical protein	0.26
ARTHROv3_520003	putative SAM-dependent methyltransferase	0.26
ARTHROv3_5240003	rph Ribonuclease PH	0.26
ARTHROv3_5650001	hmuO heme oxygenase	0.26
ARTHROv3_630014	Pentapeptide repeat protein	0.26
ARTHROv3_810074	conserved hypothetical protein	0.26
ARTHROv3_1220009	Two component transcriptional regulator	0.25
ARTHROv3_1510040	conserved hypothetical protein	0.25
ARTHROv3_4700001	conserved protein of unknown function	0.25
ARTHROv3_670001	conserved protein of unknown function	0.25
ARTHROv3_690057	ppiC Peptidylprolyl isomerase PpiC-type	0.25
ARTHROv3_840108	putative thiosulfate:cyanide sulfurtransferase (rhodanese). GlpE-like	0.25
ARTHROv3_870026	putative lactoylglutathione lyase	0.25
ARTHROv3_1240006	gvpN Gas vesicle protein GvpN	0.24
ARTHROv3_1320005	conserved protein of unknown function	0.24
ARTHROv3_1890002	putative LL-diaminopimelate aminotransferase	0.24
ARTHROv3_690145	conserved hypothetical protein	0.24
ARTHROv3_760039	conserved hypothetical protein	0.24
ARTHROv3_1130018	argD bifunctional Acetylornithine aminotransferase and Succinyldiaminopimelate transaminase	0.23
ARTHROv3_1130113	conserved protein of unknown function	0.23

ARTHROv3_1190003	btpA Photosystem I biogenesis protein btpA	0.23
ARTHROv3_1310017	Putative band 7 family protein	0.23
ARTHROv3_320028	conserved hypothetical protein (secreted)	0.23
ARTHROv3_380029	conserved hypothetical protein	0.23
ARTHROv3_4510001	rpsH 30S ribosomal subunit protein S8	0.23
ARTHROv3_630011	ctpA1 Carboxyl-terminal protease. C-terminal processing peptidase-2. Serine peptidase.	0.23
ARTHROv3_650001	garR tartronate semialdehyde reductase	0.23
ARTHROv3_650003	conserved protein of unknown function	0.23
ARTHROv3_860004	nusA transcription termination/antitermination. L factor (N utilization substance protein A)	0.23
ARTHROv3_1050032	conserved protein of unknown function	0.22
ARTHROv3_1110020	rpsA2 30S ribosomal protein S1	0.22
ARTHROv3_1300026	putative universal stress protein	0.22
ARTHROv3_130052	conserved hypothetical protein	0.22
ARTHROv3_1620077	Hydrophobic protein	0.22
ARTHROv3_170005	Alpha/beta hydrolase fold protein	0.22
ARTHROv3_210036	conserved hypothetical protein (expressed)	0.22
ARTHROv3_250010	conserved hypothetical protein	0.22
ARTHROv3_1130074	hemK Protein methyltransferase HemK	0.21
ARTHROv3_1170016	msrA2 methionine sulfoxide reductase A (fragment)	0.21
ARTHROv3_1400069	murQ N-acetylmuramic acid 6-phosphate etherase (MurNAc-6-P etherase) 4	0.21
ARTHROv3_1490038	gpmB phosphoglycerate mutase	0.21
ARTHROv3_150013	CRISPR-associated Csm3 family protein	0.21
ARTHROv3_1530050	anti-sigma B factor	0.21
ARTHROv3_3110002	conserved hypothetical protein	0.21
ARTHROv3_5510004	conserved hypothetical protein	0.21
ARTHROv3_660004	UspA domain protein	0.21
ARTHROv3_730018	Putative glyoxalase/bleomycin resistance protein/dioxygenase	0.21
ARTHROv3_1020009	speE spermidine synthase (putrescine aminopropyltransferase)	0.2
ARTHROv3_1430062	rplI 50S ribosomal subunit protein L9	0.2
ARTHROv3_1620084	cysK3 Cysteine synthase	0.2
ARTHROv3_4000002	putative FdxN element excision controlling factor protein. XisH-like	0.2
ARTHROv3_400016	psaL Photosystem I reaction center subunit XI (PSI-L) (PSI subunit V)	0.2
ARTHROv3_630021	cheW1 CheW protein. purine-binding chemotaxis protein. Chemotaxis signal transduction protein	0.2
ARTHROv3_740009	mutT mutator protein	0.2
ARTHROv3_870019	gatA Glutamyl-tRNA(Gln) amidotransferase subunit A (Glu-ADT subunit A)	0.2
ARTHROv3_870024	conserved hypothetical protein	0.2
ARTHROv3_910020	putative bacterioferritin	0.2
ARTHROv3_930032	rplM 50S ribosomal subunit protein L13	0.2
ARTHROv3_960032	ispE 4-diphosphocytidyl-2-C-methyl-D-erythritol kinase	0.2
ARTHROv3_10034	queF NADPH-dependent 7-cyano-7-deazaguanine reductase	0.19
ARTHROv3_1270018	conserved protein of unknown function	0.19
ARTHROv3_1420024	adenylate cyclase (function located in N-terminal part)	0.19
ARTHROv3_1490027	psaF Photosystem I reaction center subunit III precursor (PSI-F)	0.19
ARTHROv3_1620020	psbB Photosystem II P680 chlorophyll A apoprotein (CP-47 protein)	0.19
ARTHROv3_2030001	ilvC Ketol-acid reductoisomerase	0.19
ARTHROv3_3150002	conserved protein of unknown function	0.19
ARTHROv3_380007	glsF Ferredoxin-dependent glutamate synthase. large subunit	0.19
ARTHROv3_390015	hemC Porphobilinogen deaminase	0.19
ARTHROv3_930081	purH Bifunctional purine biosynthesis protein purH	0.19
ARTHROv3_930089	putative RuBisCO transcriptional regulator. RbcR-like	0.19
ARTHROv3_1050034	purE N5-carboxyaminoimidazole ribonucleotide mutase	0.18
ARTHROv3_1160018	rfaE rfaE bifunctional protein	0.18
ARTHROv3_1460010	gpmI 2.3-bisphosphoglycerate-independent phosphoglycerate mutase	0.18
ARTHROv3_1530075	glyS glycine tRNA synthetase. beta subunit	0.18
ARTHROv3_1550033	conserved hypothetical protein	0.18
ARTHROv3_310019	pyrD Dihydroorotate dehydrogenase	0.18
ARTHROv3_560018	hypothetical protein	0.18
ARTHROv3_10086	thiE Thiamine-phosphate pyrophosphorylase	0.17
ARTHROv3_1130142	conserved hypothetical protein	0.17
ARTHROv3_1510036	Peptidase M1. membrane alanine aminopeptidase	0.17
ARTHROv3_310079	putative SAM-dependent methyltransferase	0.17
ARTHROv3_370014	putative glycosyltransferase. family 2	0.17
ARTHROv3_430049	conserved protein of unknown function	0.17
ARTHROv3_570003	putative two-component hybrid sensor and regulator. histidine kinase	0.17
ARTHROv3_630063	atpF ATP synthase B chain (Subunit I)	0.17
ARTHROv3_810044	phaE Poly(R)-hydroxyalkanoic acid synthase. class III. PhaE subunit	0.17
ARTHROv3_850008	valS valyl-tRNA synthetase	0.17
ARTHROv3_10068	efp Elongation factor EF-P	0.16
ARTHROv3_10080	rffG dTDP-glucose 4.6-dehydratase	0.16
ARTHROv3_1180005	ask Aspartate kinase	0.16
ARTHROv3_1220004	sdhA succinate dehydrogenase flavoprotein subunit	0.16
ARTHROv3_1300038	Peroxiredoxin	0.16
ARTHROv3_160007	gmk guanylate kinase	0.16
ARTHROv3_1620019	nrdR transcriptional repressor of nrd genes	0.16
ARTHROv3_630072	hisC Histidinol-phosphate aminotransferase	0.16
ARTHROv3_760027	conserved hypothetical protein	0.16
ARTHROv3_810043	phaC Poly(R)-hydroxyalkanoic acid synthase. class III. PhaC subunit	0.16
ARTHROv3_830019	conserved hypothetical protein	0.16

ARTHROv3_1100016	cpcF Phycocyanin alpha-subunit phycocyanobilin lyase	0.15
ARTHROv3_1130129	hom homoserine dehydrogenase (HDH)	0.15
ARTHROv3_1410018	pheT phenylalanine tRNA synthetase. beta subunit	0.15
ARTHROv3_1520004	ctpA2 carboxyl-terminal processing protease; C-terminal processing peptidase-2. Serine peptidase.	0.15
ARTHROv3_1550063	leuD 3-isopropylmalate dehydratase small subunit	0.15
ARTHROv3_1570041	metK S-adenosylmethionine synthetase	0.15
ARTHROv3_1930001	methyltransferase	0.15
ARTHROv3_1940002	conserved protein of unknown function	0.15
ARTHROv3_320027	conserved hypothetical protein	0.15
ARTHROv3_420083	ftsH3 ATP-dependent zinc-metalloprotease	0.15
ARTHROv3_4720002	trpG Anthranilate synthase component 2	0.15
ARTHROv3_730002	carboxyl-terminal protease	0.15
ARTHROv3_740049	conserved hypothetical protein	0.15
ARTHROv3_840046	gltA citrate synthase	0.15
ARTHROv3_870028	Putative biopolymer transport protein. ExbD/TolR -like	0.15
ARTHROv3_1130069	putative GUN4-like regulator	0.14
ARTHROv3_1300021	conserved hypothetical protein	0.14
ARTHROv3_1380048	conserved protein of unknown function	0.14
ARTHROv3_1540080	rpsD 30S ribosomal subunit protein S4	0.14
ARTHROv3_1550007	putative UDP-N-acetyl-D-mannosamine 6-dehydrogenase	0.14
ARTHROv3_1620085	ribonuclease III. ds RNA (fragment)	0.14
ARTHROv3_190014	putative serine protease inhibitor family protein	0.14
ARTHROv3_240016	leuS leucyl-tRNA synthetase	0.14
ARTHROv3_730045	putative NAD-dependent epimerase/dehydratase	0.14
ARTHROv3_750011	putative glycosyltransferase. group 1	0.14
ARTHROv3_760028	baeR two-component system transcriptional regulator (BaeR)	0.14
ARTHROv3_780003	conserved protein of unknown function	0.14
ARTHROv3_810094	conserved protein of unknown function	0.14
ARTHROv3_810107	pps phosphoenolpyruvate synthase	0.14
ARTHROv3_840044	ndhI NAD(P)H-quinone oxidoreductase subunit I (NAD(P)H dehydrogenase I subunit I)	0.14
ARTHROv3_840091	rpoB DNA-directed RNA polymerase subunit beta	0.14
ARTHROv3_860017	putative Histone acetyltransferase. GNAT family	0.14
ARTHROv3_1130058	transcriptional regulator. Crp-like	0.13
ARTHROv3_1300018	pdxJ pyridoxine 5'-phosphate synthase	0.13
ARTHROv3_1380036	clpB1 protein disaggregation chaperone	0.13
ARTHROv3_1430031	short chain dehydrogenase	0.13
ARTHROv3_1430053	petB Cytochrome b6	0.13
ARTHROv3_1490059	pds Phytoene dehydrogenase (Phytoene desaturase)	0.13
ARTHROv3_1530021	pyrH uridylate kinase	0.13
ARTHROv3_1530056	conserved hypothetical protein	0.13
ARTHROv3_570013	conserved protein of unknown function	0.13
ARTHROv3_610007	ABC transporter. ATP-binding protein	0.13
ARTHROv3_720002	deoC Deoxyribose-phosphate aldolase (Phosphodeoxyriboaldolase) (Deoxyriboaldolase) (DERA)	0.13
ARTHROv3_860021	Exoribonuclease II	0.13
ARTHROv3_10089	Beta-lactamase-like protein	0.12
ARTHROv3_1130147	sufC component of SufBCD complex. ATP-binding component of ABC superfamily	0.12
ARTHROv3_1570037	two-component response regulator. CheY-like receiver	0.12
ARTHROv3_2520002	pflA pyruvate formate lyase activating enzyme 1	0.12
ARTHROv3_310058	putative ATPase. AAA family	0.12
ARTHROv3_370041	ribonuclease. RnE/RnG family	0.12
ARTHROv3_810052	amyA cytoplasmic alpha-amylase	0.12
ARTHROv3_1100028	conserved hypothetical protein (secreted)	0.11
ARTHROv3_120028	mazG nucleoside triphosphate pyrophosphohydrolase	0.11
ARTHROv3_1270015	conserved protein of unknown function	0.11
ARTHROv3_1300024	folD Bifunctional protein FolD	0.11
ARTHROv3_1430003	pgm phosphoglucomutase	0.11
ARTHROv3_1540063	rpsB 30S ribosomal protein S2	0.11
ARTHROv3_1610014	conserved hypothetical protein	0.11
ARTHROv3_310025	hcp Hydroxylamine reductase. hybrid-cluster	0.11
ARTHROv3_400030	conserved hypothetical protein	0.11
ARTHROv3_690137	Thioredoxin domain	0.11
ARTHROv3_790026	conserved protein of unknown function	0.11
ARTHROv3_10135	conserved hypothetical protein	0.1
ARTHROv3_1130034	putative metalloendopeptidase. M23B subfamily	0.1
ARTHROv3_120020	putative methyltransferase	0.1
ARTHROv3_150078	putative ribonuclease Z	0.1
ARTHROv3_1540055	conserved hypothetical protein	0.1
ARTHROv3_1620013	putative CoB--CoM heterodisulfide reductase subunit B. HdrB-like	0.1
ARTHROv3_190015	tol 3'(2').5'-bisphosphate nucleotidase	0.1
ARTHROv3_200030	putative WD-40 repeats containing protein. G protein beta family	0.1
ARTHROv3_2140001	dxs 1-deoxyxylulose-5-phosphate synthase. thiamine-requiring. FAD-requiring	0.1
ARTHROv3_2440001	putative UDP-glucose 4-epimerase	0.1
ARTHROv3_490006	kaiA Circadian clock protein kaiA	0.1
ARTHROv3_630068	proS prolyl-tRNA synthetase	0.1
ARTHROv3_750041	conserved hypothetical protein	0.1
ARTHROv3_850038	nadC quinolinate phosphoribosyltransferase (nicotinate-nucleotide pyrophosphorylase)	0.1
ARTHROv3_1050006	galE UDP-glucose 4-epimerase	0.09
ARTHROv3_1140027	dnaK4 Chaperone protein DnaK	0.09

ARTHROv3_1210009	conserved hypothetical protein (membrane)	0.09
ARTHROv3_1420052	ispB octaprenyl-diphosphate synthase (Octaprenyl pyrophosphate synthetase) (OPP synthetase)	0.09
ARTHROv3_1420079	conserved hypothetical protein	0.09
ARTHROv3_150011	CRISPR-associated RAMP protein	0.09
ARTHROv3_1540045	conserved hypothetical protein	0.09
ARTHROv3_1620057	galT Galactose-1-phosphate uridylyltransferase	0.09
ARTHROv3_170006	petA Cytochrome f	0.09
ARTHROv3_340022	pdhA Pyruvate dehydrogenase E1 component subunit alpha	0.09
ARTHROv3_420018	conserved hypothetical protein	0.09
ARTHROv3_550014	Two-component response regulator	0.09
ARTHROv3_570018	UTP-glucose-1-phosphate uridylyltransferase	0.09
ARTHROv3_590006	fni Isopentenyl-diphosphate delta-isomerase	0.09
ARTHROv3_660007	thrC2 Threonine synthase	0.09
ARTHROv3_660011	trpD Anthranilate phosphoribosyltransferase	0.09
ARTHROv3_810053	hypothetical protein	0.09
ARTHROv3_810075	conserved hypothetical protein	0.09
ARTHROv3_810115	conserved protein of unknown function	0.09
ARTHROv3_840092	rpoC1 DNA-directed RNA polymerase subunit gamma	0.09
ARTHROv3_10099	conserved hypothetical protein (membrane)	0.08
ARTHROv3_1090009	zam Zam protein 3'-5' exoribonuclease. VacB and RNase II	0.08
ARTHROv3_1400043	conserved hypothetical protein	0.08
ARTHROv3_1400082	aat1 Aspartate aminotransferase	0.08
ARTHROv3_1430042	adhI Alcohol dehydrogenase class-3	0.08
ARTHROv3_1440046	sqdX glycosyltransferase (sulfolipid sulfoquinovosyldiacylglycerol biosynthesis protein)	0.08
ARTHROv3_1500025	TPR domain protein	0.08
ARTHROv3_1510057	queA S-adenosylmethionine:tRNA ribosyltransferase-isomerase	0.08
ARTHROv3_1870001	metE 5-methyltetrahydropteroyltriglutamate-homocysteine S-methyltransferase	0.08
ARTHROv3_3300001	arginine biosynthesis bifunctional protein (fragment)	0.08
ARTHROv3_420008	putative glycosyltransferase. group 1	0.08
ARTHROv3_420074	putative DegT/DnrJ/EryC1/StrS aminotransferase protein family	0.08
ARTHROv3_1220008	conserved hypothetical protein	0.07
ARTHROv3_1400027	hemL glutamate-1-semialdehyde aminotransferase (aminomutase)	0.07
ARTHROv3_1420018	hisS histidyl tRNA synthetase	0.07
ARTHROv3_150024	von Willebrand factor. type A	0.07
ARTHROv3_1530059	succinylglutamate desuccinylase/aspartoacylase	0.07
ARTHROv3_1540049	conserved hypothetical protein	0.07
ARTHROv3_1540050	putative ATPase	0.07
ARTHROv3_1610009	purA adenylosuccinate synthetase. IMP--aspartate ligase. succinoadenylic kinosynthetase	0.07
ARTHROv3_1620015	fabF1 3-oxoacyl-	0.07
ARTHROv3_1620043	pntB pyridine nucleotide transhydrogenase. beta subunit	0.07
ARTHROv3_200039	pdhC Dihydrolipoamide acetyltransferase component (E2) of pyruvate dehydrogenase complex	0.07
ARTHROv3_240028	glyA Serine hydroxymethyltransferase	0.07
ARTHROv3_3340001	opcA Putative glucose 6-phosphate dehydrogenase assembly protein	0.07
ARTHROv3_350008	conserved hypothetical protein	0.07
ARTHROv3_390021	rca Ribulose bisphosphate carboxylase/oxygenase activase	0.07
ARTHROv3_4820001	murD UDP-N-acetylmuramoylalanine-D-glutamate ligase	0.07
ARTHROv3_5800006	putative Signal transduction histidine kinase (fragment)	0.07
ARTHROv3_930065	conserved protein of unknown function	0.07
ARTHROv3_10071	murE UDP-N-acetylmuramoyl-L-alanyl-D-glutamate:meso-diaminopimelate ligase	0.06
ARTHROv3_1050021	icd Isocitrate dehydrogenase	0.06
ARTHROv3_1130098	pepA cytosol aminopeptidase	0.06
ARTHROv3_120011	ugd UDP-glucose 6-dehydrogenase	0.06
ARTHROv3_1310022	Putative Phosphoglucomutase/phosphomannomutase	0.06
ARTHROv3_1440002	conserved hypothetical protein	0.06
ARTHROv3_1440029	ggpS Glucosylglycerol-phosphate synthase (Glucosyl-glycerol- phosphate synthase)	0.06
ARTHROv3_1540019	panC/cmk Bifunctional pantothenate synthetase / cytidylate kinase	0.06
ARTHROv3_1540053	conserved hypothetical protein	0.06
ARTHROv3_1610034	tldD putative peptidase	0.06
ARTHROv3_3020001	CopG-like DNA-binding (fragment)	0.06
ARTHROv3_3340002	zwf glucose-6-phosphate dehydrogenase	0.06
ARTHROv3_840068	gatB Aspartyl/glutamyl-tRNA(Asn/Gln) amidotransferase subunit B (Asp/Glu-ADT subunit B)	0.06
ARTHROv3_1010007	putative metalloendopeptidase. M16 family	0.05
ARTHROv3_130015	putative Thiazoline oxidase/Subtilisin-like protease. PatG-like	0.05
ARTHROv3_1360004	Methyltransferase type 11	0.05
ARTHROv3_1400065	ureC Urease subunit alpha (Urea amidohydrolase subunit alpha)	0.05
ARTHROv3_1400099	thrS threonyl-tRNA synthetase	0.05
ARTHROv3_1430035	sepJ putative SepJ (FraG) protein (membrane)	0.05
ARTHROv3_200041	putative long-chain-fatty-acid-CoA ligase. AMP dependent	0.05
ARTHROv3_20019	coxA Cytochrome c oxidase subunit I	0.05
ARTHROv3_2210001	reverse transcriptase-like protein; CP4-6 prophage (modular protein)	0.05
ARTHROv3_230002	prfC peptide chain release factor RF-3	0.05
ARTHROv3_240047	ilvB Acetolactate synthase large subunit	0.05
ARTHROv3_240055	putative Na+/H+ antiporter	0.05
ARTHROv3_620012	conserved protein of unknown function	0.05
ARTHROv3_880003	putative Vesicle-fusing ATPase	0.05
ARTHROv3_1140029	pilB type 4 fimbrial biogenesis protein	0.04
ARTHROv3_1210007	ppk Polyphosphate kinase	0.04
ARTHROv3_1490053	conserved hypothetical protein :	0.04

ARTHROv3_250008	nrdA Ribonucleoside-diphosphate reductase subunit alpha	0.04
ARTHROv3_310020	conserved protein of unknown function	0.04
ARTHROv3_4790005	WD-40 repeat protein (fragment)	0.04
ARTHROv3_630022	Methyl-accepting chemotaxis protein	0.04
ARTHROv3_700005	conserved hypothetical protein	0.04
ARTHROv3_870005	putative signal transduction diguanylate kinase	0.04
ARTHROv3_930073	fusA1 protein chain elongation factor EF-G. GTP-binding	0.04
ARTHROv3_1130013	FHA modulated ABC efflux pump with fused ATPase and integral membrane subunits	0.03
ARTHROv3_1170040	conserved hypothetical protein	0.03
ARTHROv3_1530058	putative helicase	0.03
ARTHROv3_240001	putative ATPase (AAA+ superfamily)	0.03
ARTHROv3_240012	alaS alanyl-tRNA synthetase	0.03
ARTHROv3_470005	carB Carbamoyl-phosphate synthase large chain	0.03
ARTHROv3_630074	NTPase (NACHT family)-like protein	0.03
ARTHROv3_1300041	TPR repeat containing protein (tetratricopeptide)	0.01
