# Supplementary material for: Proteome-Wide Analysis and Diel Proteomic Profiling of the Cyanobacterium Arthrospira platensis PCC 8005
Source: PLoS One. 2014 Jun 10;9(6):e99076. doi: 10.1371/journal.pone.0099076 (PMC4051694; doi:10.1371/journal.pone.0099076)
Supplement: Table S4 — List of proteins identified in the flowthrough and elution factions of the Cu-IMAC experiment (107 proteins). (PDF) [file pone.0099076.s004.pdf]

Table S4. List of identified proteins found in common in both the flowthrough and elution factions of the Cu-IMAC experiment (107 proteins)

Accession number	Name of proteins	empAI values	
		Flowthrough	Eluate
ARTHROv3_6210004	Allophycocyanin beta subunit	345.89	23.30
ARTHROv3_5910003	Allophycocyanin alpha chain (fragment)	99.31	4.89
ARTHROv3_430053	apcD Allophycocyanin alpha-B subunit	29.59	5.56
ARTHROv3_1100010	cpcB C-phycocyanin beta subunit	9.66	8
ARTHROv3_1100011	cpcA C-phycocyanin alpha subunit	8.74	22.39
ARTHROv3_380023	psbV Cytochrome c-550 precursor (Cytochrome c550) (Low-potential cytochrome c)	4.42	0.97
ARTHROv3_1420023	Orange carotenoid-binding protein	4.37	0.56
ARTHROv3_1430028	putative peroxiredoxin	3.82	9.58
ARTHROv3_230011	ahpC alkyl hydroperoxide reductase. C22 subunit	3.14	0.53
ARTHROv3_390003	sodB superoxide dismutase. Fe	3.08	1.02
ARTHROv3_1280008	apcF allophycocyanin beta-18 subunit	2.81	1.31
ARTHROv3_10081	petJ Cytochrome c6 (Soluble cytochrome f) (Cytochrome c553)	2.04	0.74
ARTHROv3_760031	groS Cpn10 chaperonin GroES	2.01	1.29
ARTHROv3_1530080	conserved hypothetical protein	1.89	9.93
ARTHROv3_810066	putative Calvin cycle regulator CP12-like protein	1.8	14.62
ARTHROv3_1890003	trxB Thioredoxin-2	1.72	2.49
ARTHROv3_1100014	cpcD Phycobilisome 8.9 kDa linker polypeptide. phycocyanin-associated. rod	1.49	2.38
ARTHROv3_1540042	psbO photosystem II manganese-stabilizing polypeptide precursor (MSP)	1.31	0.69
ARTHROv3_760032	groL2 Cpn60 chaperonin GroEL. large subunit of GroESL	1.28	0.64
ARTHROv3_680013	hypothetical protein	1.2	0.37
ARTHROv3_430055	fabG2 3-oxoacyl-	1.1	3.4
ARTHROv3_1460006	psaE Photosystem I reaction center subunit IV	0.97	0.4
ARTHROv3_1620014	acpP acyl carrier protein	0.96	0.4
ARTHROv3_1510065	conserved hypothetical protein	0.9	8.46
ARTHROv3_1440012	conserved hypothetical protein (secreted)	0.85	0.36
ARTHROv3_1910001	hup3 Histone-like bacterial DNA-binding protein. HU-like	0.83	0.35
ARTHROv3_870032	bcp4 Bacterioferritin comigratory protein	0.8	9.54
ARTHROv3_1210010	dnaK3 Chaperone protein DnaK	0.75	0.39
ARTHROv3_620015	tig trigger factor	0.73	0.13
ARTHROv3_760001	apcE Phycobilisome core-membrane linker polypeptide (Anchor polypeptide LCM)	0.66	0.71
ARTHROv3_10067	accB Biotin carboxyl carrier protein of acetyl-CoA carboxylase	0.63	0.18
ARTHROv3_1380010	conserved hypothetical protein	0.62	0.38
ARTHROv3_1130035	conserved hypothetical protein	0.59	1.94
ARTHROv3_680022	conserved hypothetical protein	0.59	1.01
ARTHROv3_50002	conserved hypothetical protein	0.53	0.24
ARTHROv3_5240002	conserved hypothetical protein	0.52	0.11
ARTHROv3_760017	putative Transcriptional regulator. AbrB/MazE/MraZ-like	0.48	6.21
ARTHROv3_420082	conserved protein of unknown function	0.47	0.47
ARTHROv3_4780004	rplS 50S ribosomal subunit protein L19	0.47	0.47
ARTHROv3_780005	conserved protein of unknown function	0.47	2.21
ARTHROv3_6210003	apcC Phycobilisome 7.8 kDa linker polypeptide. allophycocyanin-associated. core (LC 7.8)	0.45	2.07
ARTHROv3_690010	conserved hypothetical protein	0.43	1.04
ARTHROv3_640021	conserved protein of unknown function	0.4	0.96
ARTHROv3_530005	conserved protein of unknown function	0.38	0.62
ARTHROv3_760047	Putative PpiC-type peptidyl-prolyl cis-trans isomerase	0.38	5.98
ARTHROv3_360008	putative nucleoside-diphosphate-sugar epimerase	0.34	0.06
ARTHROv3_1420011	ftsZ cell division protein; tubulin-like GTP-binding protein and GTPase	0.33	0.9
ARTHROv3_960039	conserved hypothetical protein	0.29	0.09
ARTHROv3_240054	putative hydrolase	0.29	0.07
ARTHROv3_250003	putative two-component system transcriptional regulator. LuxR family	0.26	0.12
ARTHROv3_300043	clpB2 protein disaggregation chaperone	0.26	1.67
ARTHROv3_570058	hypothetical protein	0.26	1.16
ARTHROv3_1520008	putative metalloendopeptidase (peptidase M23B)	0.25	0.25
ARTHROv3_370048	tuf protein chain elongation factor EF-Tu	0.24	3.77
ARTHROv3_5570001	Translation initiation factor IF-2 (fragment)	0.22	0.09
ARTHROv3_1240001	protein of unknown function	0.21	1.18
ARTHROv3_1050068	protein of unknown function	0.2	0.15
ARTHROv3_2320001	atpG ATP synthase gamma chain	0.2	0.57
ARTHROv3_2080002	conserved protein of unknown function	0.19	0.19
ARTHROv3_630061	atpA F1 sector of membrane-bound ATP synthase. alpha subunit	0.19	0.19
ARTHROv3_1420067	groL1 Cpn60 chaperonin GroEL. large subunit of GroESL	0.18	0.92
ARTHROv3_1490020	ilvH Acetolactate synthase small subunit	0.18	0.91
ARTHROv3_1050022	recA DNA strand exchange and recombination protein with protease and nuclease activity	0.17	0.5
ARTHROv3_1320006	conserved protein of unknown function	0.16	0.16

ARTHROv3_1400143	ppa inorganic pyrophosphatase	0.16	0.16
ARTHROv3_1450034	conserved hypothetical protein	0.16	1.99
ARTHROv3_1610010	rplY 50S ribosomal protein L25 (General stress protein CTC)	0.16	0.16
ARTHROv3_430009	conserved protein of unknown function	0.16	0.34
ARTHROv3_750014	NADPH-dependent FMN reductase	0.16	0.16
ARTHROv3_1090012	clpP4 proteolytic subunit of ClpA-ClpP and ClpX-ClpP ATP-dependent serine proteases	0.15	0.15
ARTHROv3_160006	conserved hypothetical protein	0.15	0.23
ARTHROv3_1380007	msrA1 methionine sulfoxide reductase A	0.14	0.67
ARTHROv3_1540064	tsf protein chain elongation factor EF-Ts	0.14	4.98
ARTHROv3_1410001	conserved protein of unknown function	0.13	0.09
ARTHROv3_1140028	grpE Protein grpE (HSP-70 cofactor)	0.12	1.16
ARTHROv3_1160014	putative Haloacid dehalogenase-like hydrolase	0.12	0.12
ARTHROv3_200001	conserved hypothetical protein	0.12	2.62
ARTHROv3_400029	pgl 6-phosphogluconolactonase (6PGL)	0.12	0.26
ARTHROv3_4190001	conserved protein of unknown function	0.12	0.12
ARTHROv3_590035	ccmM carbon dioxide concentrating mechanism protein	0.11	0.92
ARTHROv3_910016	putative phycobilisome linker polypeptide	0.11	1.03
ARTHROv3_630018	iaaA L-asparaginase. Peptidase T2. asparaginase 2. Isoaspartyl peptidase/ dipeptidase)	0.1	0.61
ARTHROv3_1100013	cpcC2 Phycobilisome 32 kDa linker polypeptide. phycocyanin-associated. rod 2	0.1	1.9
ARTHROv3_1210005	psbD1 Photosystem II D2 protein (PSII D2 protein) (Photosystem Q(A) protein)	0.09	0.08
ARTHROv3_1470032	gap2 Glyceraldehyde-3-phosphate dehydrogenase 2	0.09	0.41
ARTHROv3_1070008	gap1 Glyceraldehyde-3-phosphate dehydrogenase 1	0.09	0.19
ARTHROv3_1140026	dnaJ Chaperone protein DnaJ	0.08	0.08
ARTHROv3_1320007	Endoribonuclease L-PSP	0.08	0.08
ARTHROv3_320004	fbaA Fructose-bisphosphate aldolase	0.08	0.5
ARTHROv3_430033	yjgB putative alcohol dehydrogenase. Zn-dependent and NAD(P)-binding	0.08	1.08
ARTHROv3_930104	psaA Photosystem I P700 chlorophyll a apoprotein A1 (PsaA)	0.08	0.08
ARTHROv3_1130139	hypothetical protein	0.07	0.25
ARTHROv3_960030	eno enolase	0.07	0.32
ARTHROv3_1210012	atpD ATP synthase subunit beta	0.06	3.32
ARTHROv3_260008	conserved hypothetical protein	0.06	0.13
ARTHROv3_420092	cbbL Ribulose bisphosphate carboxylase large chain (RuBisCO large subunit)	0.06	1.59
ARTHROv3_1530051	two-component response regulator	0.05	0.11
ARTHROv3_2890001	protein chain elongation factor EF-G. GTP-binding (fragment)	0.05	0.94
ARTHROv3_930083	tktA transketolase 1. thiamin-binding	0.05	0.85
ARTHROv3_930103	psaB Photosystem I P700 chlorophyll a apoprotein A2 (PsaB)	0.04	0.12
ARTHROv3_1130103	clpC ATP-dependent Clp protease regulatory subunit	0.04	1.09
ARTHROv3_3000001	protein disaggregation chaperone (fragment)	0.04	0.52
ARTHROv3_250011	ileS Isoleucyl-tRNA synthetase	0.03	0.06
ARTHROv3_200011	protein of unknown function	0.02	0.01
ARTHROv3_200022	Periplasmic protein TonB links inner and outer membranes-like protein	0.02	0.02
ARTHROv3_70003	conserved hypothetical protein	0.02	1.82
ARTHROv3_1510023	Protein kinase of the HstK subfamily (with both a Ser/Thr kinase domain and a His kinase domain)	0.01	0.01
